# Carbon starvation, senescence and specific mitochondrial stresses, but not nitrogen starvation and general stresses, are major triggers for mitophagy in Arabidopsis

**DOI:** 10.1080/15548627.2022.2054039

**Published:** 2022-04-03

**Authors:** Sylwia M. Kacprzak, Olivier Van Aken

**Affiliations:** Department of Biology, Lund University, Lund, Sweden

**Keywords:** Arabidopsis, autophagy, mitochondria, mitophagy, plants, senescence

## Abstract

Selective degradation of mitochondria by autophagy (mitophagy) is thought to play an important role in mitochondrial quality control, but our understanding of which conditions induce mitophagy in plants is limited. Here, we developed novel reporter lines to monitor mitophagy in plants and surveyed the rate of mitophagy under a wide range of stresses and developmental conditions. Especially carbon starvation induced by dark-incubation causes a dramatic increase in mitophagy within a few hours, further increasing as dark-induced senescence progresses. Natural senescence was also a strong trigger of mitophagy, peaking when leaf yellowing became prominent. In contrast, nitrogen starvation, a trigger of general autophagy, does not induce strong increases in mitophagy. Similarly, general stresses such as hydrogen peroxide, heat, UV-B and hypoxia did not appear to trigger substantial mitophagy in plants. Additionally, we exposed plants to inhibitors of the mitochondrial electron transport chain, mitochondrial translation and protein import. Although short-term treatments did not induce high mitophagy rates, longer term exposures to uncoupling agent and inhibitors of mitochondrial protein import/translation could clearly increase mitophagic flux. These findings could further be confirmed using confocal microscopy. To validate that mitophagy is mediated by the autophagy pathway, we showed that mitophagic flux is abolished or strongly decreased in *atg5/AuTophaGy 5* and *atg11* mutants, respectively. Finally, we observed high rates of mitophagy in etiolated seedlings, which remarkably was completely repressed within 6 h after light exposure. In conclusion, we propose that dark-induced carbon starvation, natural senescence and specific mitochondrial stresses are key triggers of mitophagy in plants.

**Abbreviations:** AA: antimycin A; ATG: AuToPhagy related; ConA: concanamycin A; DIS: dark-induced senescence; Dox: doxycycline; FCCP: carbonyl cyanide-p-trifluoromethoxyphenylhydrazone; GFP: green fluorescent protein; IDH1: isocitrate dehydrogenase 1; MB: MitoBlock-6; Mito-GFP: transgenic Arabidopsis line expressing a mitochondrially targeted protein fused to GFP; mtETC: mitochondrial electron transport chain; OXPHOS: oxidative phosphorylation; PQC: protein quality control; TOM20: Translocase of Outer Membrane 20

## Introduction

Mitochondria are double-membrane organelles essential for all living organisms, as they govern cellular energy metabolism and maintain cellular homeostasis. This includes production of energy in the form of ATP through oxidative phosphorylation (OXPHOS), synthesis of metabolic precursors or metabolic by-products, like heme or iron-sulfur clusters [1]. Unfortunately, mitochondria are also inevitably highly prone to proteotoxic stresses, which can be toxic to the cells [2]. One prominent reason is the generation of reactive oxygen species (ROS), as a natural by-product from the unpaired electrons during electron transfer at complexes I–III of the respiratory chain [3,4]. The mitochondrial derived ROS can increase due to organelle dysfunctions or external stresses, leading to the degradation of mitochondrial proteins, lipids and DNA [3,5]. Furthermore, due to their endosymbiotic origin, mitochondrial proteins can be encoded by the nuclear and mitochondrial genomes [6,7]. The gene expression of the two genomes is therefore tightly regulated, to reduce possibility for the accumulation of unassembled respiratory chain units [8,9]. To ensure mitochondrial proteostasis, all eukaryotes have developed protein quality control (PQC) systems, which involve various proteolytic degradation or repair pathways [5,10–12]. These are required for the recognition and elimination of the individually misfolded proteins, protein aggregates, or the whole dysfunctional mitochondria, as well to balance mitochondrial protein import with the cellular demands.

In all eukaryotes, the major pathways of the mitochondrial PQC include: the mitochondria-to-nucleus transcriptional communication, termed mitochondrial unfolded protein response (UPR^mt^; [13,14]), the cytosolic multi protein ubiquitin-proteasome degradation machinery (UPS; [15]), and mitophagy, a catabolic process where damaged mitochondria are selectively delivered by the autophagy machinery to the vacuole/lysosomes for degradation [16]. Another important mechanism for mitochondrial PQC conserved in all eukaryotes relies on the removal of damaged organellar proteins and regulation of mitochondrial protein activity by a series of mitochondrial adenosine triphosphate (ATP) – driven proteases, like plant mitochondrial serine proteases Lon, Clp and FtsH metalloproteases [17]. Selective autophagy of mitochondria is one of the most intensively studied autophagy mechanisms in yeast and animal systems. It is critical not only for maintaining cellular homeostasis, but additionally serves in recycling cellular energy and resources, with strong implications for whole organism health and fitness [18,19]. The mitophagy process requires recognition of the damaged organelles by specific receptors that recruit mitochondria as their cargo, and incorporate them into growing autophagy structures. During autophagy, receptors with their cargo are initially surrounded by a double membranous, cup-shaped, structure called a phagophore, which expands and closes into double-membrane vesicle (an autophagosome). Ultimately, autophagosomes fuse with the vacuole (yeast and plants) or lysosomes (animals), which allows to degrade and break down their cargos by a wide set of hydrolases, while remaining molecules can be released back for reuse [20,21].

Formation of the autophagy machinery involves concerted action of multiple autophagy-related (ATG) proteins, initially characterized in yeast, but well conserved in metazoans [22] and plants [21]. Of these, ubiquitin-like protein ATG8 appears as fundamental. Not only does it coordinate all steps of phagophore and autophagosome formation by forming membrane-bound conjugates with its lipidated form, but ATG8 isoforms can also interact with various cargo receptors via a conserved motif called LC3-interacting region (LIR), or Atg8-interacting motif (AIM) [23], and therefore providing a mechanism for selective autophagy. Several, mitochondria-localized mitophagy receptors have been identified, like the yeast Atg32 protein [24], or in mammals PINK1, NIPSNAP1, NIPSNAP2, FUNDC1, BNIP3L/NIX, BNIP3, BCL2L13, and PHB2 [25–29]. Whereas mitochondria in metazoans and plants share functional similarity, plants lack orthologs for mitophagy receptors or regulators present in other organisms [30]. To date, plant specific mitophagy receptors remain poorly understood. It has been suggested that plant ATG11 is recruited as an adaptor during mitochondrial degradation via autophagy [31]. Recently, Ma et al. [32], demonstrated that FMT/Friendly Mitochondria/Friendly, a mostly cytosolic protein controlling mitochondrial division in plants [33], may also be a regulator of plant mitophagy. Loss of FMT results in large aggregates of clustered mitochondria [34], which resulted in incomplete autophagosome formation around the mitochondria. Although a wide range of conditions have been demonstrated to trigger mitophagy in yeast or mammals including hypoxia [26], uncoupler treatments [35], chemical inhibitors of mitochondrial ATP synthase (oligomycin) and electron transport chain (antimycin A) [36], oxidative stresses [37], or various diseases [38], the role of mitophagy in plants is not well understood. The limited reports demonstrate that plant mitochondria can undergo specific autophagic degradation during nutrient starvation [31,32], or when *Arabidopsis* plants are treated with protonophore 2,4-dinitrophenol (DNP) that causes mitochondrial membrane depolarization [32].

One of the major complications for studying mitophagy in plants is the lack of efficient and reliable mitophagy monitoring systems. To date, observations of mitophagy in plants have been carried out using time-consuming methods that require specific and expensive equipment, such as electron microscopy and confocal microscopy [32,39]. Furthermore, these microscopy-based methods are not very suitable for obtaining (semi-)quantitative information about the rates of mitophagy and are prone to observer bias. In this study we established an efficient and semi-quantitative system to monitor mitophagic flux, by exploiting the inherent resistance of the globular green fluorescent protein (GFP) to acidic degradation in e.g. vacuoles and lysosomes [24]. By fusing GFP to mitochondrially-targeted proteins or ATG8, and measuring the relative abundance of full-length GFP-fusion protein and free GFP via immunodetection, we can specifically monitor the relative rate of mitophagy and general autophagy, respectively.

Currently, a clear overview of which environmental and developmental conditions trigger mitophagy in plants is lacking. Therefore, we used our reporter system to screen a range of conditions that are known or suspected to induce general autophagy or mitophagy in e.g. yeast and animal systems. We screened various plant stressors that are specific to the mitochondria, or have broad effects on the whole cell. We additionally support our findings with live cell imaging microscopy to visualize autophagosome or mitophagosome accumulation. We show that mitophagy in plants is predominantly induced during prolonged exposure to darkness and carbon starvation, irrespective of the plant age, and during natural senescence. In contrast, nitrogen starvation and many abiotic stresses do not appear to be efficient inducers of mitophagy in plants. We also show that plants and yeast/animals only partially share similarity in the induction of mitophagy by mitochondrial stressors. Our work thus provides new insights into the specificity and molecular control of the mitophagy process in plants.

## Results

### Establishment of a system to specifically monitor mitophagy in plants

**Figure 1.**
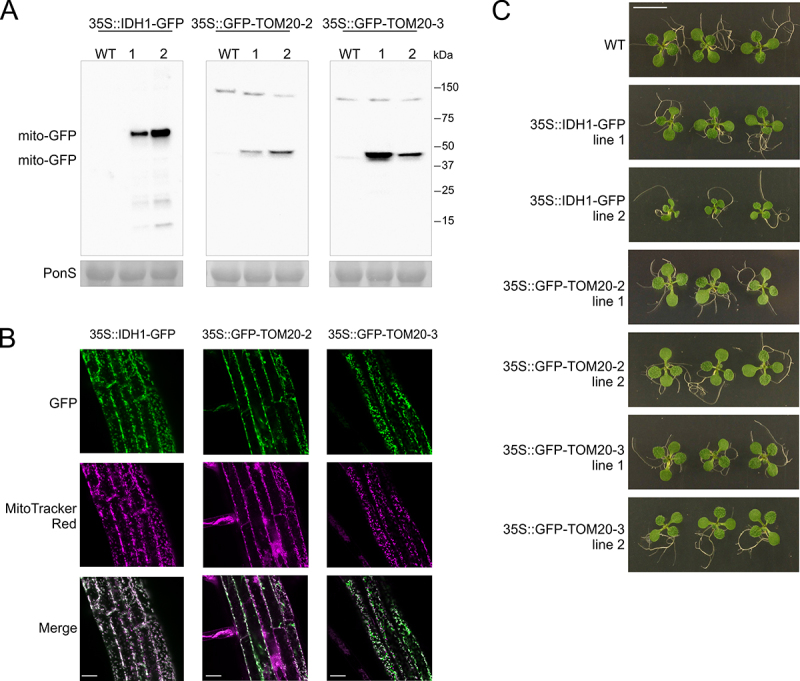
Generation of stable *Arabidopsis* transgenic lines for measuring mitophagy in plants. *IDH1, TOM20-2* and *TOM20-3* were expressed under the control of the *35S* promoter as a fusion with the GFP fluorescent protein, in the wild type (WT) Col-0 line. (A) Western blot analysis of GFP-fusion proteins IDH1-GFP, GFP-TOM20-2 and GFP-TOM20-3 in 35S::IDH1-GFP, 35S::GFP-TOM20-2 and 35S::GFP-TOM20-3 transgenic lines. All lines, including control WT (Col-0), were grown for 10 days on 1/2 MS agar medium with 1% sucrose. Equal loading is indicated by Ponceau S-stained Rubisco large subunit (RBCL)(PonS, bottom panel). (B) Confirmation of mitochondrial localization of fusion proteins in the root cells of 7-d-old *Arabidopsis* seedlings. IDH1-GFP, GFP-TOM20-2 and GFP-TOM20-3 fluorescence signals were visualized by fluorescence confocal microscopy. GFP (green) fluorescence was recorded alongside the mitochondrial marker MitoTracker Red (magenta). Overlay of both fluorescence channels is shown in the merged image. Scale bars: 20 µm. (C) Representative phenotype of 10-d-old *Arabidopsis* seedlings of two independent homozygous lines (line 1, 2) grown together with the WT (Col-0) control on 1/2 MS agar medium supplemented with 1% sucrose. Scale bar: 7 mm.

Mitophagy has been monitored in e.g. yeast by fusing mitochondrially-targeted proteins to GFP, and observing the accumulation of the degradation-resistant free GFP as a measure of mitophagic flux [24]. To establish such a system to monitor mitophagy in plants, we produced stable *Arabidopsis thaliana* (Arabidopsis) transformants that express GFP fusion proteins with either the mitochondrial matrix-localized IDH1 (isocitrate dehydrogenase 1; *35S::IDH1-GFP*) or mitochondrial outer membrane localized TOM20 (Translocase of Outer Membrane 20; *35S::GFP-TOM20-2* and *35S::GFP-TOM20-3*). Presence of the expression constructs was confirmed by PCR on genomic DNA (Figure S1A) and steady-state levels of the chimeric proteins were examined using SDS-PAGE followed by western blotting with an anti-GFP antibody (Figure 1A). For all constructs, two independently transformed lines were selected, with intermediate to high levels of the GFP fusion proteins (collectively referred to as “mito-GFP” lines). Full-length GFP-fusion proteins were observed in all lines, whereas under these optimal growth conditions no free GFP (molecular mass 26.9 KDa) could be observed, suggesting very low levels of mitophagy in young plants under growth permissive conditions. Some nonspecific bands could be observed after longer exposures, but these did not appear at the expected size of free GFP. Correct mitochondrial targeting of the three fusion proteins was verified by confocal microscopy using MitoTracker Red as a marker for mitochondria (Figure 1B). In general, the produced transgenic Arabidopsis plants showed a similar growth phenotype as wild type Col-0 plants (Figure 1C). Only in the *35S::IDH1-GFP* line 2 a clear growth reduction was observed, which is in line with its high levels of IDH1-GFP fusion protein (Figure 1A).

**Figure 2.**
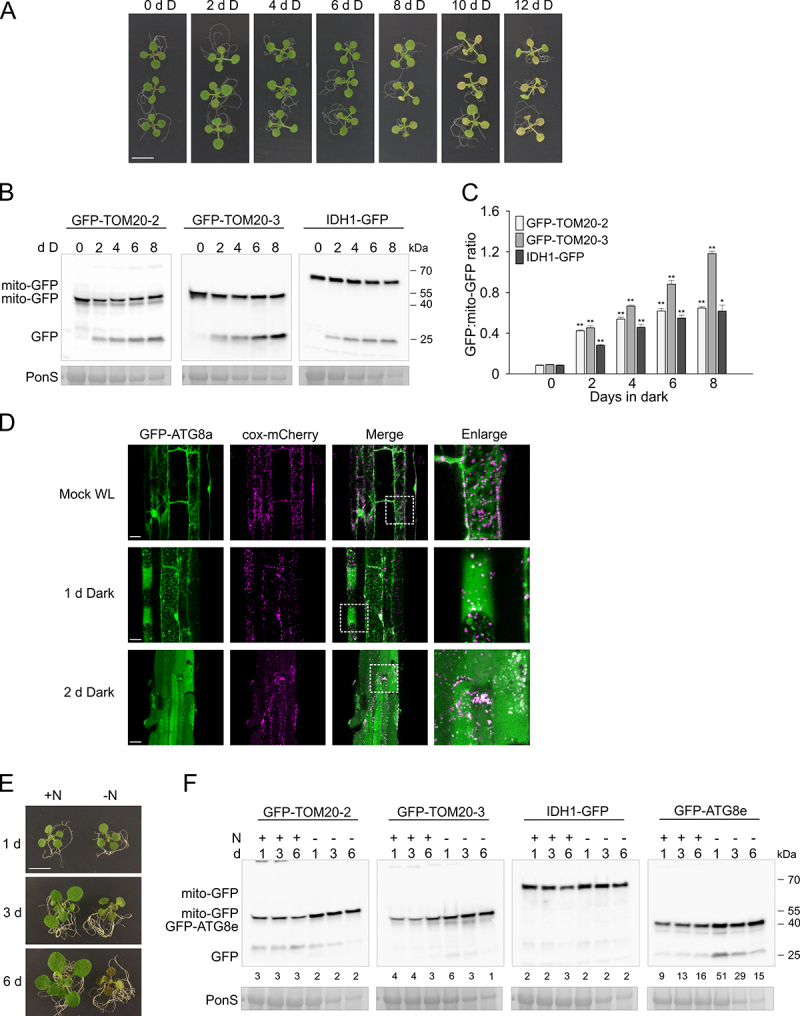
Dark induced carbon starvation, but not N starvation, induces mitophagy in *Arabidopsis*. (A) Representative images for the WT (Col-0) de-greening phenotypes under carbon starvation (dark-induced senescence). 10-d-old *Arabidopsis* seedlings grown on 1/2 MS agar medium, without sucrose under 16/8 h photoperiodic light conditions were transferred to constant darkness for 2–12 days. Scale bar: 7 mm. (B) Immunoblot detection of GFP cleavage in three “mito-GFP” transgenic lines at indicated time points (days) during carbon starvation treatment using anti-GFP antibody. “Mito-GFP” indicates the full length GFP fusion proteins, “GFP” indicates the free GFP moiety. RBCL stained with Ponceau S was used as a loading control (PonS, bottom panels). (C) Bar charts illustrating ratios of free GFP to full-length “mito-GFP” in (B). Bars shown are the means (± SE) of three biological replicates (Student’s *t*-test; *p < 0.05, **p < 0.01). Asterisks denote a significant difference versus respective mitochondrial-GFP line before transferring to darkness (0 days in dark). (D) Colocalization of mitochondria with GFP-ATG8a autophagosomes in root cells of the *Arabidopsis* transgenic line expressing *35S::GFP-ATG8a* and *35S::cox-mCherry* analyzed by fluorescence confocal microscopy. Autophagosome formation in the dark was induced by transferring **7**-d-old seedlings grown vertically to liquid 1/2 MS supplemented with 0.5 μM concanamycin A (ConA) and incubation for 24 h in the dark (1 d Dark), or transferring seedlings from agar medium into darkness for 48 h in liquid 1/2 MS, with 0.5 μM ConA added for the last 1 6 h before imaging (2 d Dark). Scale bars: 20 µm. (E) Example images for WT (Col-0) phenotypes under N starvation. 10-d-old *Arabidopsis* seedlings grown on 1/2 MS medium, supplemented with 1% sucrose were transferred to the 1/2 MS medium without N source (Merck; M0529) and left to continue growth under the standard 16/8 h photoperiodic light conditions for 1–6 days. Scale bar: 7 mm. (F) Representative western blot images of IDH1-GFP, GFP-TOM20-2, GFP-TOM20-3 and GFP-ATG8e fusion proteins, or free GFP detection, using anti-GFP antibody at indicated time points after N starvation treatment. Numerical values represent relative intensity ratios (%) of free GFP to GFP-mito or GFP-ATG8e. RBCL from total protein lysates stained with Ponceau S was used as a loading control (PonS, bottom panels).

Autophagy has been strongly associated with carbon starvation in plants, which can easily be applied by depriving plants of light while growing on media without a carbon source [40,41]. Indeed, when 10-day-old wild-type seedlings grown on MS media without sucrose were moved into the dark, clear signs of leaf yellowing could be observed from day 6 onward, with near complete bleaching after 10–12 days (Figure 2A). To assess whether mitophagy occurs during this dark-induced senescence (DIS), we exposed the mito-GFP reporter lines to a similar regime and measured GFP-cleavage by immunodetection (Figure 2B). Very clear accumulation of free GFP could be observed in three “mito-GFP” reporter lines already in samples at day 2, with increasing amounts of free GFP polypeptide accumulating toward day 8. In contrast, the overall protein content per mg of fresh tissue of the samples reduced over time as indicated by Ponceau and Coomassie stainings (Figure 2B, Figure S2A). The relative abundance of both full-length protein and free GFP was quantified from the immunoblots and a strong increase in the GFP:mito-GFP ratio was observed already at day 2 and continued to increase further in a time-dependent manner toward day 8 (Figure 2C).

As mitophagy is often detected using confocal microscopy, we wanted to confirm that our immunodetection system is indeed representative for mitophagy using a similar method. Therefore, we created a transgenic line which expresses both the previously characterized *GFP-ATG8a* [40] general autophagy reporter and a mitochondrially targeted mCherry (*cox-mCherry*). The plants were pretreated with concanamycin A (ConA), which inhibits vacuolar acidification and therefore blocks proteolysis of autophagosome content, to allow visual identification of autophagosomes. When grown under normal “mock” white light conditions, the GFP-ATG8a protein could be observed around the cell membrane and diffusely throughout the cell (Figure 2D). After transferring seedlings to darkness for one day, clear GFP-ATG8a autophagic puncta could be observed in root cells. Upon closer examination, overlaps of some of these GFP-ATG8a puncta with the cox-mCherry mitochondrial marker could be seen. After two days in darkness, GFP-ATG8a puncta that overlapped with mitochondria were even more apparent, further supporting that DIS induces high rates of mitophagy and that the immunodetection-based detection system of mitophagy is representative for microscopical observations.

### Nitrogen starvation and general abiotic stresses do not induce high rates of mitophagic flux

A wide range of stresses induce general autophagy, and have been reported to induce mitophagy in yeast and animal systems, and for some stresses such as N-starvation and UV-B also in plants [32,39]. Therefore, we used our transgenic reporter lines and immunoblot GFP protein detection system to monitor the induction of mitophagy under a range of conditions. Nitrogen starvation has been a classic way to induce bulk autophagy also in plants [40], but only milder nitrogen starvation-induced recycling of mitochondrial proteins was reported previously [32]. In order to investigate long term effects of N starvation, without pleiotropic stress effects, like hypoxia, we used an agar plate transfer system instead of liquid culture growth (see methods for detail). After three days on -N media, the plants had hardly grown, and by day six the plants looked very pale and stressed (Figure 2E), and showed reduced protein content (Figure S2B). In contrast, control plants transferred to +N media continued to grow vigorously (Figure 2E). In agreement with previous reports, even short exposure to one day of N starvation triggered increased GFP cleavage of the ATG8 family member ATG8e GFP fusion protein (GFP-ATG8e), compared to control conditions, indicating general autophagy activation (Figure 2F). In contrast, no free GFP could be observed in any of the three mitophagy reporter lines. Only a weak unspecific band could occasionally be observed at a higher MW than free GFP, as also observed in Figure 2B. To further examine whether mitochondria are not targeted for autophagic degradation during N starvation, we monitored colocalization of mitochondria with ATG8a by confocal fluorescence microscopy, using seedlings co-expressing *cox-mCherry* and *GFP-ATG8a*. Under standard conditions (mock), we observed diffuse cytosolic GFP-ATG8a signals that unspecifically overlapped with mitochondrial fluorescence (Figure S3A). However, when incubated for 24 h in -N media with ConA, multiple GFP-ATG8a puncta appeared in the mesophyll cells, which did not clearly colocalize with cox-mCherry signal (Figure S3A).

**Figure 3.**
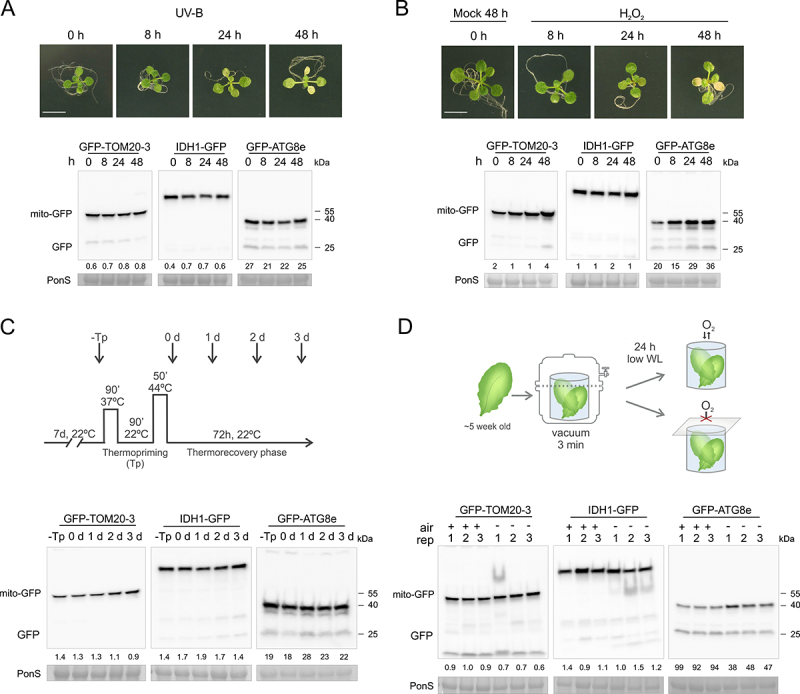
Mitophagy is not induced by various general stresses. IDH1-GFP, GFP-TOM20-3, GFP-ATG8e fusion proteins, and free GFP were detected by immunoblotting using an anti-GFP antibody, after UV-B stress (A), hydrogen peroxide (H_2_O_2_) treatment (B), heat stress recovery (C) or hypoxia (D). For each representative blot, Ponceau S-stained RBCL from total protein lysates was included as loading control (PonS, bottom panels). (A-B) 10-d-old seedlings grown on 1/2 MS medium with 1% sucrose were exposed to 10,000 mJ cm^−2^ UV-B (A) or sprayed with 100 mM H_2_O_2_ (B) and returned to standard growth conditions. Plant photographs at the top panels, show representative seedlings 8–48 h after the stress treatments. Scale bars: 7 mm. (C) Top panel indicates schematic representation of temperature regimes applied to study heat stress (HS). 7-d-old seedlings were exposed to mild HS at 37°C, returned to optimal temperature at 22°C for recovery followed by high HS at 44°C. Seedlings were additionally returned to standard growth conditions for 1–3 days for the prolonged HS recovery phase. (D) Top panel shows schematic representation of the multi-well plate experimental set up, applied to probe hypoxia stress. Whole leaves of 5-week-old plants were gently immersed in 24-well plates filled with assay medium, vacuum-treated in a desiccator for 3 min to remove residual air from intracellular tissue spaces, and sealed with transparent film to block O_2_. Control samples were treated the same, except plates were left unsealed throughout 24 h of incubation. Relative intensity ratios (% of free GFP:GFP-mito or GFP-ATG8e) for all abiotic stresses are shown as numeric values below blots.

Past reports show that UV-B stress in plants can induce selective autophagy (chlorophagy [42];), and triggers increased colocalization of mitochondria with autophagic puncta [39]. Therefore, we exposed 10-day-old seedlings to a UV-B dose of 10,000 mJ/s^−1^/cm^−1^ and monitored GFP processing at different times after recovery, using the same set of reporter lines. Already after 24 h some damage could be observed in the plants, with clear leaf chlorosis 48 h after the UV-B stress, indicating the treatment was effective (Figure 3A). Some free GFP polypeptide accumulation could be observed in the GFP-ATG8e line even under control conditions, but no increase was noted during the 48 h time course. Similarly, no clear change in GFP cleavage from the mitochondrially targeted reporters could be observed. Consistent with the immunoblot analysis, we could not observe a clear increase in appearance of GFP-ATG8a puncta in mesophyll cells of the *cox-mCherry GFP-ATG8a* line by fluorescence confocal microscopy, within two days after exposure to UV-B stress (Figure S3A, lower panel). Nonetheless, the fluorescence imaging revealed increased accumulation of clustered mitochondria in UV-B stressed seedlings, a phenotype reported previously [39]. Overall, in our hands and growth conditions, N-starvation and UV-B do not appear to be strong inducers of mitophagy in plants.

Reactive Oxygen Species (ROS) are closely associated with stress response and signaling in plants, and have been proposed to induced general autophagy in plants [20,43]. To test if H_2_O_2_ can specifically induce mitophagy, Arabidopsis seedlings were sprayed with 100 mM H_2_O_2,_ and GFP cleavage was assessed at a range of time points (Figure 3B). After 48 h, clear signs of damage, like leaf chlorosis and loss of turgor, could be observed in the cotyledons, indicating the treatment induced oxidative stress. The treatment induced an increase in overall GFP-ATG8e protein level, indicating the H_2_O_2_ stress did not inhibit protein synthesis. Nevertheless, a clear increase in free GFP polypeptide in the samples from GFP-ATG8e line was observed from 24 h, indicating an increased general autophagic flux. No consistent signs of free GFP were observed in the immunoblots of the *GFP-TOM20-3* and *IDH1-GFP* lines, suggesting mitophagy is not strongly induced by H_2_O_2_ in this time frame.

We next examined if recovery from heat stress (HS), here referred to as “thermopriming”, can induce mitophagy, using an experimental set-up described in the diagram in Figure 3C and adopted from Sedaghatmehr et al., 2016 [44]. Immunoblots of extracts from heat-primed mito-GFP, and control *GFP-ATG8e* 7d old seedlings with anti-GFP antibody show no significant increase in free GFP, both right after priming or during 3 days of the recovery phase (Figure 3C). Although some changes in accumulation of the full chimeric proteins were detected, e.g. higher levels of GFP-TOM20-3 at days 2–3 of thermorecovery, these were not consistent between all 3 lines examined. To assess whether individual mitochondria can colocalize with autophagosomes during recovery from the HS, we visualized *cox-mCherry GFP-ATG8a* seedlings 2 d after thermopriming (Figure S3B). Under primed conditions, we occasionally observed GFP-ATG8a puncta, while only diffuse cytosolic GFP-ATG8a signal appeared in control untreated samples. However, among samples from primed plants, we could not detect colocalization of GFP-ATG8a foci with mitochondrial fluorescence signals (Figure S3B). Therefore, also here no convincing evidence for mitophagy could be observed.

Finally, the effect of low oxygen on mitophagy was assessed using a procedure similar to [45], where leaves collected from 5-week-old plants were rolled up and submerged in multiwell-plates (Figure 3D, top panel). We incubated plates with or without oxygen access under continuous low light for 24 h to avoid photosynthetic oxygen production, though not full darkness (as commonly implemented for hypoxia stress assays), to avoid induction of mitophagy by dark exposure (Figure 2A). Though some increase in full-length GFP-ATG8e protein could be observed under low oxygen conditions, the level of free GFP remained stable. Similarly, no evidence for elevated free GFP levels was observed in the mito-GFP lines after the hypoxia treatment. To verify that our treatment procedure successfully caused hypoxia, we analyzed O_2_ depletion in a similar plate assay using an O_2_-sensitive fluorescent dye. As expected, we observed a gradual and significant increase in relative fluorescence within approximately 6 h after sealing, while fluorescence in unsealed samples remained stable (Figure S4A). We also quantified mRNA levels of 3 genes known to be induced during low oxygen stresses [45] (Figure S4B). All 3 genes (*ADH1, PGB1* and *PDC1*) were significantly upregulated 24 h after sealing, relative to untreated samples and samples incubated for 24 h in the open wells. To further examine autophagosome and mitochondrial localization during low oxygen stress, we visualized *cox-mCherry GFP-ATG8a* plants following the experimental set-up described above (Figure S3C). The GFP-ATG8a protein was mostly diffused within the cytosol. Occasionally, we observed formation of individual ATG8a puncta. These were however present to a similar extent both in seedlings incubated in control (opened) and low oxygen (sealed) wells with assay medium supplemented with ConA. The observed GFP-ATG8e puncta were thus probably triggered by the low light treatment, but unlinked to the hypoxia. Under both conditions, we could not detect colocalization of the GFP-ATG8a puncta with mitochondria. We repeated immunoblot detection of GFP in 12-d-old seedlings exposed to low oxygen by incubating agar plates in anaerobic jars for 24 h (Figure S4C). Similarly as before, no significant increase of the free GFP band was observed in hypoxia-stressed samples as compared to controls (aerobic), in both mito-GFP and GFP-ATG8e lines. We further confirmed induction of the hypoxia marker genes in seedlings incubated in anaerobic jars, indicating the plants suffered from low oxygen stress (Figure S4D). Together, these findings indicate that general abiotic stresses such as UV-B, H_2_O_2_, heat and hypoxia are not strong inducers of mitophagic flux, or general autophagy (except H_2_O_2_), in plants.

### Specific mitochondrial stresses trigger mitophagy in plants

**Figure 4.**
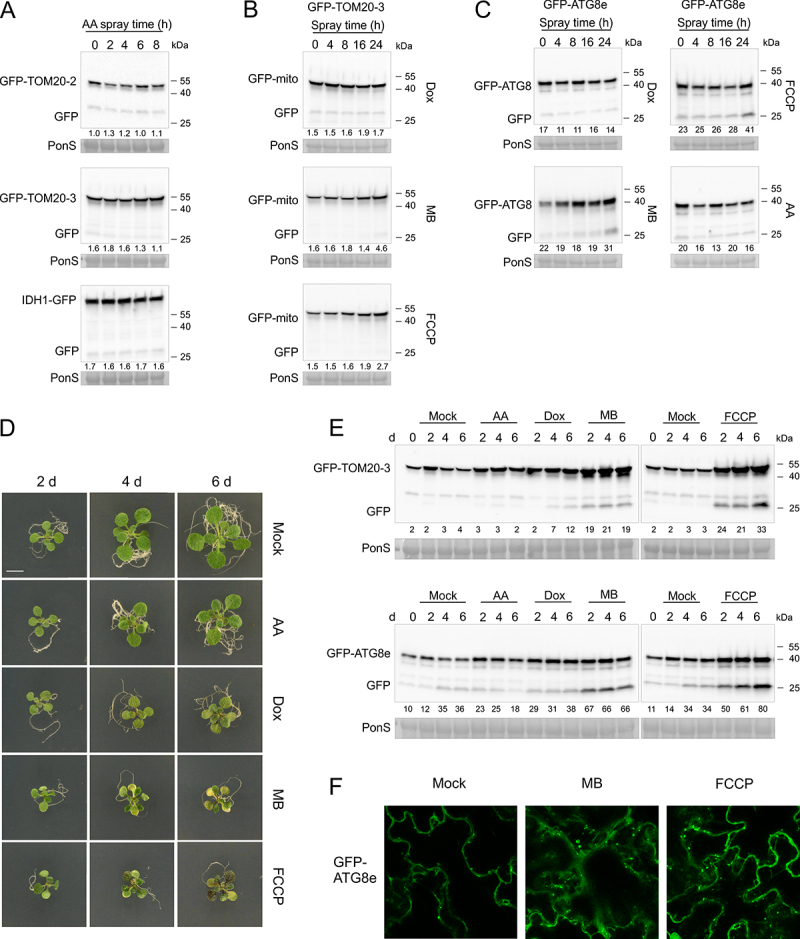
FCCP and MB mitochondrial stress treatments efficiently induce mitophagy and general autophagy in *Arabidopsis*. (A-C) Immunoblot detection of GFP cleavage in 10-d-old three “*mito-GFP”* lines, or *GFP-ATG8e* line at indicated time points after spray with 50 μM antimycin A (AA), 25 μg/ml doxycycline (Dox), 50 μM MitoBloCK-6 (MB), or 20 μM carbonyl cyanide-4 (trifluoromethoxy) phenylhydrazone (FCCP), using anti-GFP antibody. Numerical values represent relative intensity ratios (%) of free GFP to GFP-mito (A-B), or GFP to GFP-ATG8e (C). RBCL stained with Ponceau S served as an equal loading control (PonS, bottom panels). (D) Representative images of phenotypes of *GFP-TOM20-3* line after exposure to mitochondrial inhibitor stresses for prolonged time. 10-d-old seedlings were germinated on 1/2 MS medium supplemented with 1% sucrose, followed by transfer to fresh growth media supplemented with 30 μM AA, 15 μg/ml Dox, 10 μM MB, or 10 μM FCCP. Seedlings were grown on mito inhibitors under the same light conditions for additional 2–6 days. Scale bar: 5 mm. (E) Immunoblot detection of fusion proteins: GFP-TOM20-3 (top panel), GFP-ATG8e (bottom panel), and free GFP, using anti-GFP antibody at indicated time points after transfer to various mitochondrial inhibitors as shown in (D). Relative intensity ratios (% of free GFP:GFP-mito or GFP-ATG8e) are shown as numerical values. Equal total protein loading is indicated by Ponceau S-stained RBCL (PonS) (bottom panels). (F) Confocal fluorescence microscopy-based detection of GFP-ATG8e autophagosome formation in cells of abaxial cotyledon epidermis upon longer exposure to mitochondrial protein import inhibitor (MB) or mitochondrial uncoupler (FCCP). 7-d-old seedlings were transferred to fresh agar medium, supplemented with, or without (Mock), 10 μM MB, or 10 μM FCCP in 24-well plate wells and left to growth for an additional 3 days. 24 h before imaging, plants were incubated under constant white light with additional 300 μL of liquid 1/2 MS with 1 μM ConA, added to each well to allow autophagosome visualization. Scale bars: 10 μm.

As mitophagy is thought to be involved in mitochondrial quality control, we assessed whether a range of mitochondrial inhibitors could increase mitophagic flux in plants. We selected four chemicals that inhibit mitochondrial function at different functional sites, including antimycin A (AA; Complex III), doxycycline (Dox; mitochondrial ribosomes), carbonyl cyanide-p-trifluoromethoxyphenylhydrazone (FCCP; uncoupler) and MitoBlockCK-6 (MB; mitochondrial protein import). In previous work, these chemicals have been sprayed onto the plants or added to the growth media, where they can induce rapid upregulation of mitochondrial retrograde/unfolded protein response (UPR^mt^) target gene expression and inhibit plant growth [46,47]. First, we tested spraying of in vitro grown 10-day-old mito-GFP seedlings with the four chemicals. Despite its well-described effects on mitochondrial OXPHOS, no clear cleavage of the mito-GFP full length proteins could be observed up to 8 h after AA spray (Figure 4A). Spraying with Dox also did not result in clear free GFP accumulation up to 24 h after spraying (Figure 4B). In contrast, spraying with FCCP and MB did result in a weak, but reproducible accumulation of free GFP in the *GFP-TOM20-3* line, indicating mild activation of mitophagy (Figure 4B). Both, MB and FCCP also induced accumulation of free GFP in the GFP-ATG8e reporter line, especially at the end of the 24 h time course, indicating that these chemicals also induce moderate levels of general autophagy (Figure 4C).

Next, we transferred 10-day-old seedlings from normal MS media to fresh agar media supplemented with the four mitochondrial inhibitors, so we could monitor autophagic flux over a longer time scale. The negative effects on plant growth could clearly be observed during the following days, with AA having the mildest effects and MB/FCCP showing the strongest inhibition of plant growth, with cotyledon chlorosis developing at days 4–6 post transfer (Figure 4D). The activation of mitophagy by prolonged growth on mitochondrial inhibitors was assessed using immunoblots of protein extracts prepared from the *GFP-TOM20-3* line. The results were overall similar to those obtained with inhibitor spraying, but the effects of longer exposure to MB and FCCP were much more pronounced, with clear accumulation of free GFP after 2–6 days (Figure 4E). Some mild GFP processing could also be observed after 4–6 days of growth on Dox. Interestingly, free GFP-cleavage on mitochondrial inhibitors was also accompanied by higher accumulation of full-length GFP-TOM20-3 fusion protein, over the time course. The chemical inhibitor treatments had no strong effect on the total protein accumulation (Figure S2C). Densitometric analysis of the relative amount of free GFP vs full-length GFP could nevertheless clearly show that Dox, and especially MB and FCCP, could induce increased mitophagic flux (Figure 4E, Figure S5A). In agreement with the induction of mitophagy by selected mitochondrial inhibitors, an accumulation of free GFP was observed in the *GFP-ATG8e* line after MB and FCCP treatment, indicating that these chemicals activate general autophagy and mitophagy (Figure 4E and Figure S5B). To further validate whether FCCP and MB could indeed activate autophagy, we examined *GFP-ATG8e* seedlings using confocal microscopy three days after transfer to the media containing mitochondrial inhibitors, with additional pre-incubation with ConA to allow visualization of autophagosomal puncta (Figure 4F). As expected, MB or FCCP treated seedlings accumulated much greater number of GFP puncta in cotyledon mesophyll cells, as compared to the mock treated samples, in which GFP-ATG8 signal was mostly diffused (Figure 4F). In conclusion, mitochondrial inhibition in plants does not seem to result in wide-spread induction of mitophagy, though specific inhibitors that depolarize mitochondria (FCCP), interfere with protein import (MB) or translation (Dox) can activate selective mitophagy.

### Chemical activators and inhibitors of autophagy affect mitophagy in plants

**Figure 5.**
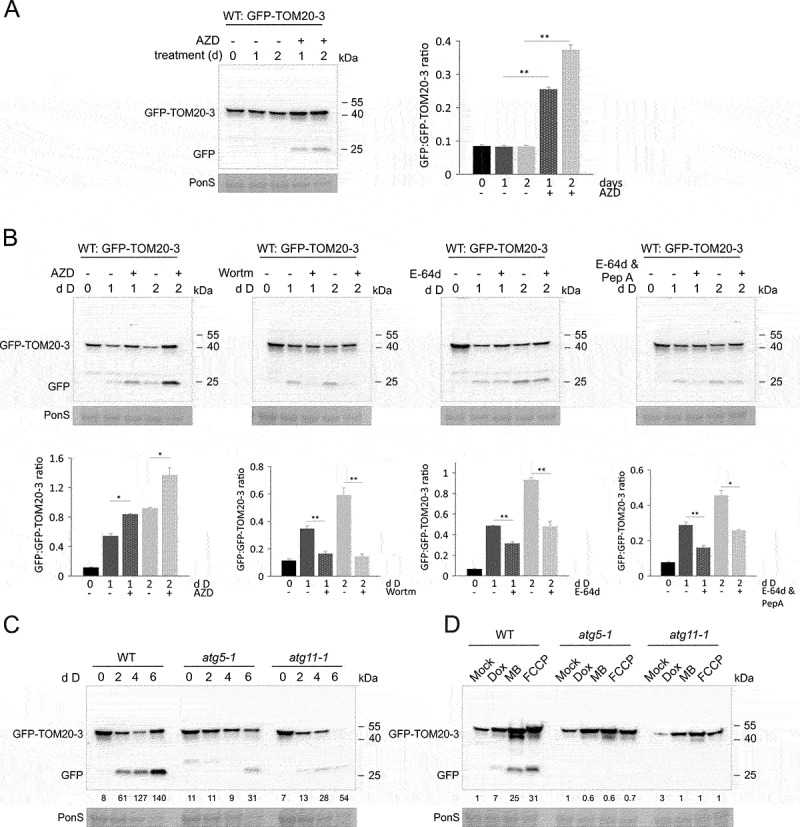
Mitophagy induced during carbon starvation or growth on mitochondria inhibitors is blocked in autophagy mutants, or by chemical autophagy inhibitors. (A) GFP cleavage assay for mitophagy induction by TOR chemical inhibition. 7-d-old GFP-TOM20-3 seedlings grown vertically were transferred to liquid 1/2 MS media, supplemented with 10 μM AZD8055 (AZD) or DMSO and left under Standard light growth conditions for 1 to 2 days. Immunoblot detection of GFP-TOM20-3 and free GFP was performed with anti-GFP antibody. Bar chart on the right represents means (± SE) for free GFP to GFP-TOM20-3 ratios from three independent experiments (Student’s *t*-test; **p < 0.01). (B) Top panel: Immunoblot detection of GFP cleavage of dark treaded WT seedlings expressing GFP-TOM20-3 using anti-GFP antibody. Total protein extracts were prepared from 7-d-old vertically grown seedlings, incubated for 1–2 additional days in the dark in liquid 1/2 MS media, supplemented with or without 10 μM AZD8055 (AZD), or 5 μM wortmannin (Wortm), or 20 μM E-64d with or without additional supplementation with pepstatin A (PepA). Bottom panel: bar charts illustrating ratios of free GFP to GFP-TOM20-3 from the immunoblots in the top panel. Bars shown are the means (± SE) of three biological replicates (Student’s *t*-test; *p < 0.05, **p < 0.01). Asterisks denote a significant difference versus seedlings incubated in the dark, without various inhibitors. (C) GFP cleavage assay for carbon starvation (dark-induced senescence) in WT, *atg5-1* and *atg11-1* seedlings expressing mitochondrial fusion protein GFP-TOM20-3. 10-d-old seedlings grown on 1/2 MS agar medium without sucrose (pH 5.7) were transferred to constant darkness for 2–6 days. Full-length fusion protein and free GFP were detected by immunoblot using anti-GFP antibody. Control Ponceau S stain of RBCL was used to visualize changes in total protein content from lysates upon dark treatment (PonS, bottom panel). (D) GFP cleavage assay of WT, *atg5-1* and *atg11-1 GFP-TOM20-3* seedlings exposed to mitochondrial chemical inhibitors: Dox, MB and FCCP, for 4 days, using the same experimental system as described in Figure 5D. Presence of the full-length fusion protein and free GFP was confirmed by immunoblot detection with anti-GFP antibody. Ponceau S stained RBCL (PonS) from total protein lysates, was used as a loading control. Relative intensity ratios (% of free GFP:GFP-TOM20-3) on carbon starvation (C) or mitochondrial inhibitors (D) are shown as numeric values.

Many chemical treatments have been shown to induce or block autophagy by affecting various functional sites in the autophagy pathway in plants and other organisms. To further validate that the immunoblot GFP cleavage-based assays are representative for autophagy, we transferred 7-d-old *GFP-TOM20-3* seedlings to liquid media supplemented with AZD8055, an inhibitor of the protein kinase TOR (target of rapamycin [20],), which is a key negative regulator of autophagy. Within 1 d of incubation under standard light growth conditions, we observed a clear and significant accumulation of free GFP, which was even stronger after 2 d of treatment, in contrast to mock treated controls where no free GFP band was detected (Figure 5A). This indicates that the inhibition of TOR leads to constitutive mitophagy. Subsequently, we repeated the GFP cleavage assay with AZD8055 treatment under dark, to identify additive effects with carbon starvation-dependent autophagy (Figure 5B). Free GFP was detected in GFP-TOM20-3 samples incubated in the dark, and its amount was higher when AZD8505 was added, especially after 2 d in the dark. Although the amount of full-length GFP-TOM20-3 protein also increased with AZD8505 supplementation, the free GFP:GFP-TOM20-3 ratios were still higher in the presence of AZD8505 as compared to mock samples for the same time point.

Next, we tested if known inhibitors of autophagy could also block mitophagy in plants. Therefore, we treated GFP-TOM20-3 plants with wortmannin, a phosphatidylinositol 3-kinase (PI3K) inhibitor that blocks autophagosome formation [20], and collected samples after 1 and 2 days in the dark (Figure 5B). As expected, the dark treatment alone induced clear TOM20-3 GFP processing. In contrast, no free GFP was detected in immunoblots when the dark-treated plants had been incubated with wortmannin (Figure 5B). Wortmannin-induced inhibition of PI3Ks can thus effectively block mitophagy in plants.

Vacuolar protein degradation during autophagy can be inhibited using the cysteine protease inhibitor E-64d [48]. To further validate that the release of free GFP in our reporter system is mediated by vacuolar protease activity, we performed a follow-up DIS immunoblot experiment on the *GFP-TOM20-3* plants incubated in the dark, with or without E-64d supplemented to the medium (Figure 5B). Both at day 1 and day 2 of darkness, it could be observed that E-64d reduced degradation of the full-length GFP-TOM20-3 protein. Some free GFP accumulation could still be observed, suggesting that the applied concentration of E-64d did not fully block vacuolar protein degradation. When GFP-TOM20-3 seedlings were incubated in the dark in medium supplemented with E-64d in combination with aspartyl protease inhibitor pepstatin A (Pep A), accumulation of free GFP was much more reduced as compared to the mock treated samples, especially at day 1 (Figure 5B), confirming that the free GFP represents vacuolar degradation. The ratios of free GFP:GFP-TOM20-3 were significantly lower in the presence of E-64d solely or with addition of Pep A, indicating the importance of vacuolar proteases for the mitochondrial processing.

### Darkness- and mitochondrial inhibition-induced mitophagy requires ATG5 and ATG11

As autophagy is genetically controlled by ATG proteins, we wanted to test if the observed induction of mitophagic flux by dark-induced senescence or specific mitochondrial inhibitors is indeed dependent on the ATG pathway. To do this, we produced mutants in ATG5 (a general autophagy regulator) and ATG11 (implicated in mitophagy) [31] that express the *35S::GFP-TOM20-3* construct. The validity of the *atg5-1* and *atg11-1* mutations and presence of the GFP-mitochondria construct were verified by PCR on genomic DNA (Figure S1B). The plants were also exposed to extended darkness, and the accelerated senescence phenotype was observed very clearly and at a similar level in all *atg5-1* mutant lines, and to a more moderate extent in all *atg11-1* mutants (Figure S1C), as previously described [31,40].

To assess the role of ATG5 and ATG11 in mitophagy, as well as to validate that the immunoblot GFP cleavage-based assays are representative for true autophagy, we first exposed 10-day-old wild type (WT), *atg5-1* and *atg11-1* lines expressing the *GFP-TOM20-3* construct to extended darkness. In the WT background, clear accumulation of free GFP was observed already after two days in the dark (Figure 5C). In contrast, accumulation of free GFP was completely abolished at day 2–4 in the *atg5-1* mutants (Figure 5C). Similarly, only very low levels of free GFP were observed in the *atg11-1* background after transfer to darkness. We conclude that ATG5 plays a critical role in DIS-induced mitophagy in plants, and ATG11 is not essential but contributes to it.

The role of ATG proteins during mitophagy induced by mitochondrial inhibitors was also assessed using the *atg5-1* and *atg11-1* lines expressing the *35S::GFP-TOM20-3* construct. 10-day-old plants of the different genotypes were transferred onto media containing DOX, MB or FCCP for 4 days and GFP processing was assessed by immunodetection (Figure 5D). In the wild type background, clear accumulation of free GFP could be observed especially with FCCP and MB, while Dox resulted in a weaker accumulation. Again, an overall accumulation of the full length GFP-TOM20-3 protein could be observed as before (Figures 4E and 5D). In contrast, no free GFP could be detected in the *atg5-1* or *atg11-1* backgrounds for any of the three mitochondrial inhibitors tested. Interestingly, the accumulation of full length GFP-TOM20-3 after inhibitor treatment was also reduced in the *atg* mutants as compared to the WT line, suggesting that mitochondrial quality control by mitophagy is required to result in accumulation of the GFP-TOM20-3 protein. In summary, our results show that mitophagy is controlled by ATG5 and ATG11 proteins in plants.

### Natural (age-dependent) senescence induces mitophagy in plants

**Figure 6.**
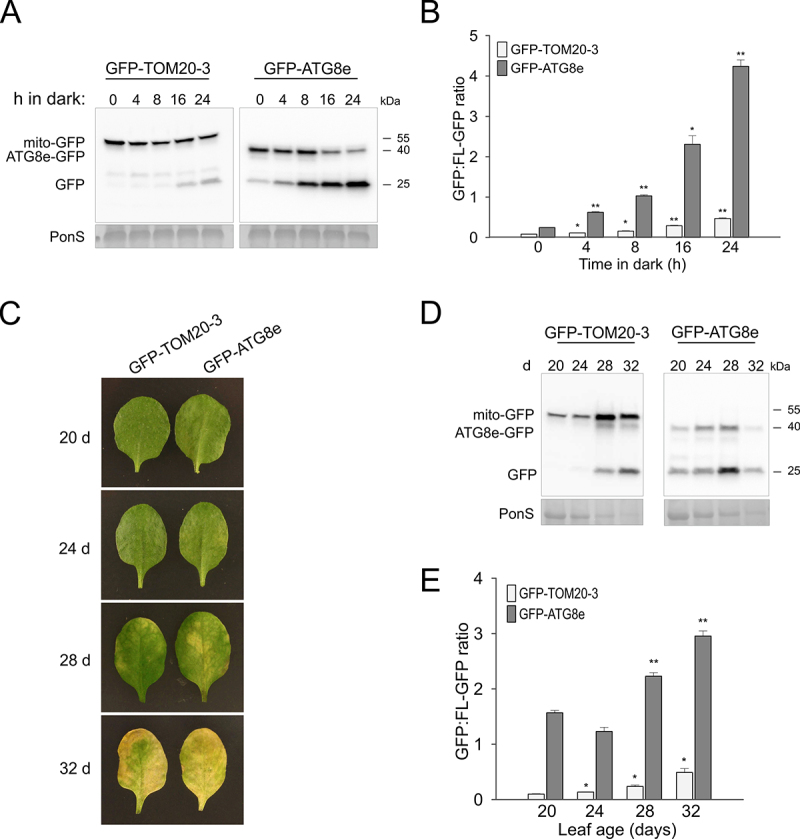
Mitophagy is induced during short exposure to darkness, as well during natural senescence. (A) Immunoblot detection of GFP cleavage in WT GFP-TOM20-3 and GFP-ATG8e transgenic lines at indicated early time points during carbon starvation treatment, using anti-GFP antibody. 10-d-old seedlings grown on 1/2 MS agar medium under the 16/8 h photoperiodic light conditions were transferred to constant darkness for up to 24 h. Total protein levels of the lysate are indicated by Ponceau S stained RBCL (PonS; bottom panel). (B) Bar charts illustrating ratios of free GFP to GFP-TOM20-3 or GFP-ATG8e in (A). Bars shown are the means (± SE) of three biological replicates (Student’s *t*-test; *p < 0.05, **p < 0.01). Asterisks denote a significant difference versus respective GFP fusion line under control light conditions (0 h in dark). (C) Representative photographs of the 4-th rosette leaf of *GFP-TOM20-3* and *GFP-ATG8e* transgenic lines, grown in the soil under the 16/8 h photoperiodic light conditions for indicated time. (D) Immunoblot detection of GFP cleavage in WT *GFP-TOM20-3* and *GFP-ATG8e* transgenic lines at different age stage during natural senescence (as defined in C), using anti-GFP antibody. Changes in the total protein content from protein lysates were indicated by Ponceau S stain (PonS; bottom panel). (E) Bar charts illustrating ratios of free GFP to GFP-TOM20-3 or GFP-ATG8e in (D). Asterisks denote a significant difference versus respective line from the earliest time point (20-d-old leaf) (Student’s *t*-test; *p < 0.05, **p < 0.01).

Our results indicate that DIS and associated carbon starvation are among the strongest triggers of mitophagy in plants, with very clear accumulation of free GFP and autophagic puncta after 1–2 days in the dark (Figure 2). To assess how fast darkness can induces mitophagy, we performed a short darkness time course focusing on the first 24 h (Figure 6A). A very clear processing of GFP-ATG8e could be observed already at 4 h after transfer to dark, and the free GFP protein accumulation progressed subsequently within next hours. A clear free GFP band was also rapidly detected in immunoblots for samples from the *GFP-TOM20-3* line. The changes after 4–8 h were less pronounced in the *GFP-TOM20-3* line, but quantification of the immunoblot signals confirmed a significantly higher ratio of free GFP:GFP-TOM20-3 and GFP:GFP-ATG8e even after 4 h (Figure 6B). Thus, darkness can induce significant rates of mitophagy within the course of hours.

Autophagy has been strongly associated with natural age-related senescence [49,50]. To assess if senescence is also a trigger of mitophagy in plants, we monitored free GFP-TOM20-3 and GFP-ATG8e processing in soil grown plants without any treatment. Leaf 3 and 4 were taken as a representative to monitor natural senescence and sampled from 20 days after seedling emergence (when the leaves are still healthy and green) up to day 32 (when leaf senescence and loss of chlorophyll is very evident) (Figure 6C). While no free GFP accumulation could be observed in the *GFP-TOM20-3* line in the healthy green leaf (day 20), first signs of GFP processing were detected in immunoblots at day 24, which became progressively stronger the more visible senescence could be observed in the plants (Figure 6D). Levels of full-length GFP-TOM20-3 also increased at day 28–32, although overall protein content per mg of fresh weight of tissue dropped dramatically (Figure 6D, Figure S2E). This is in agreement with the fact that chloroplasts are degraded first during senescence, while mitochondria remain present and active until the last moments of senescence [51]. Therefore, it is expected that mitochondrial proteins represent a larger fraction of the total protein content during late senescence. Regardless, the ratio of free GFP:GFP-TOM20-3 increased steadily from day 24 to day 32 (Figure 6E), indicating mitophagy is strongly activated during natural senescence in plants. Some free GFP could already be observed in the *GFP-ATG8e* line at day 20 and the GFP:GFP-ATG8e ratio was already higher than 1, much higher than free GFP ratios in young GFP-ATG8e non-stressed seedlings in e.g. Figure 2F, suggesting general autophagy had already started at this time point (Figure 6D/E). A further increase in free GFP was particularly notable at day 28, while full-length GFP-ATG8e protein was nearly fully processed at day 32. Such an elevated ratio of GFP:GFP-ATG8e at day 28–32 was further validated by quantification of the immunoblot signals (Figure 6E).

### Light-exposure rapidly suppresses mitophagy in dark-germinated seedlings

**Figure 7.**
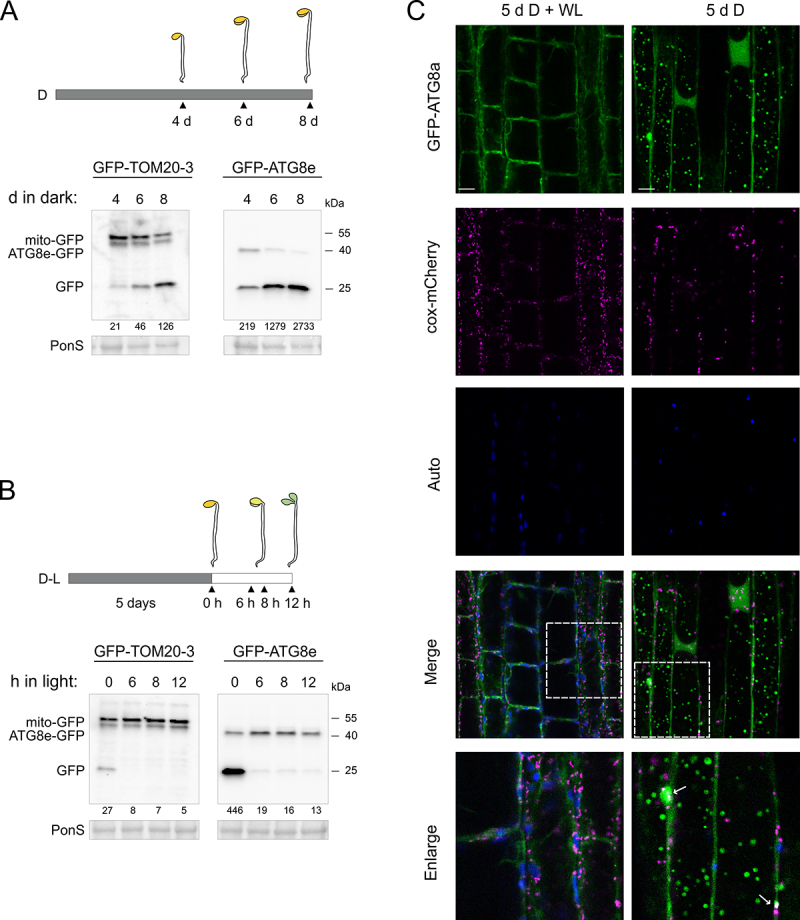
General autophagy and mitophagy are induced in etiolated seedlings in prolonged darkness, but decline rapidly after transition to white light. (A-B) GFP cleavage assay in dark etiolated (A), or de-etiolated (B) *GFP-TOM20-3* and *GFP-ATG8e* seedlings. full-length fusion proteins, and free GFP were detected by immunoblot using anti-GFP antibody. total protein content in cell lysates is indicated by Ponceau S stains (PonS). (A) Top panel indicates schematic representation of etiolation growth assay. seedlings were sown on 1/2 MS medium, exposed to white light (WL) for 2 h to induce germination, followed by a 4- up to 8 days of growth in continuous darkness. (B) Top panel shows schematic representation of de-etiolation assay, where seedlings were grown on 1/2 MS medium for 5 days in the dark and transferred to WL for 6, 8 and 12 h. numerical values represent relative intensity ratios (%) of free GFP to GFP-TOM20-3 or GFP-ATG8e (A-B). (C) Fluorescence confocal microscopy analysis of colocalization of mitochondria with GFP-ATG8a autophagosomes in hypocotyl cells of etiolated transgenic line expressing *35S::GFP-ATG8a* and *35S::cox-mCherry*. 24 h before imaging, seedlings were incubated in the liquid 1/2 MS media supplemented with 0.5 μM concanamycin A (ConA) under constant WL (left panel) or in the dark (right panel) to allow autophagosome visualization. overlay of three fluorescence channels is shown in the merged images. white arrows indicate individual mitochondria colocalizing with fluorescence signals from the round shaped GFP-ATG8a structures. scale bars: 10 µm.

The above findings clearly indicate that when plants are shifted to dark, the rate of mitophagy increases strongly. Therefore, we were interested to see how active mitophagy is in plants that have never been exposed to light. Seeds of the *GFP-TOM20-3* and *GFP-ATG8e* lines were germinated in the dark to induce skotomorphogenesis (etiolation) up to 8 days (Figure 7A). Immunodetection clearly showed free GFP in the *GFP-TOM20-3* line already at day 4, and the GFP:GFP-TOM20-3 ratio increased steadily the longer the plants were in the dark (Figure 7A, Figure S6A). A similar but even more pronounced processing was observed in the *GFP-ATG8e* line, where the vast majority of GFP-ATG8e fusion protein had been processed by 6–8 days of dark germination (Figure 7A, Figure S6A).

Given the high levels of mitophagy in etiolated seedlings, we assessed how fast mitophagy is switched off when the seedlings are exposed to light, and etioplasts transition to chloroplasts. In a similar setup, seeds of the *GFP-TOM20-3* and *GFP-ATG8e* line were germinated in the dark for 5 days. Subsequently, they were transferred to normal white light and samples were collected during the first 12 h of de-etiolation (Figure 7B). Immunodetection of free and full-length GFP confirmed that both mitophagy and general autophagy were highly active after 5 days of dark germination. Impressively, within 6 h of exposure to light, mitophagy and general autophagy had been completely suppressed, as evidenced by near-complete disappearance of free GFP in both *GFP-TOM20-3* and *GFP-ATG8e* lines (Figure 7B). To validate this result, we also implemented confocal microscopy to test for the formation of autophagosomes in the double fluorophore *GFP-ATG8e cox-mCherry* line (Figure 7C). In agreement with our western blots, clear formation of GFP-ATG8e puncta was observed in the hypocotyl cells that have been grown for 5 days in darkness (Figure 7C), and to a lesser extent in cotyledon cells (Figure S7). However, after transferring the etiolated seedlings to 24 h of constant white light, no clear sign of GFP-ATG8e puncta could be observed (Figure 7C, Figure S7). Overlaying with the cox-mCherry mitochondrial marker signals indicated that some mitochondria colocalized with GFP-ATG8e autophagic puncta. We additionally monitored autofluorescence signals to ensure specificity of the GFP detection, and to confirm that the plants were undergoing photomorphogenesis. In conclusion, mitophagy in plants appears to be closely associated with dark-growth and can be rapidly switched off by only a few hours of light exposure.

## Discussion

Mitochondrial proteostasis is an indispensable component of cellular homeostasis, cellular activity, and stress acclimation. Mitochondrial dysfunction is closely associated with reduced cellular growth and diseases [38]. Multiple PQC systems, including autophagy, are thought to have evolved to regulate and maintain organellar proteostasis. Despite the growing interest in the field of plant mitochondrial stress responses, the role for selective mitochondria recycling via autophagy remains unclear, and mechanistically it is still a relatively poorly understood process. Although autophagy has initially been identified as a bulk cellular degradation system, it is now well accepted as a highly selective quality control process for the rapid removal and recycling of toxic macromolecules [52] including whole damaged organelles, like mitochondria.

Here we aimed to better understand mitochondrial turnover via mitophagy during plant growth and interactions with the environment. We have developed a system to detect mitophagic flux in plants in an unbiased way, not fully relying on microscope analysis. We have applied this system to explore which conditions can trigger mitophagy in plants. Our findings indicate that mitophagy is not induced to high levels by a broad range of general stress conditions in plants such as heat, hypoxia, UV-B and H_2_O_2_, despite previous reports that UV-B may increase mitophagy [39]. For instance, N starvation, a classical inducer of general autophagy in plants and mitophagy in yeast [53], does not appear to induce a strong increase in mitophagy in plants. Similarly, N starvation also does not appear to be a major trigger of chlorophagy in plants [42]. Plants therefore do not seem to rely on targeted energy-organellar autophagy to compensate for a lack of externally available N. This may be because the N present in these organelles, which is fixed there in proteins crucial for the plant’s energy supply, is too important to sacrifice in the short term as mitochondrial function is required for N mobilization upon resupply [54]. As N is largely taken up by the root system and then divided across the plant depending on need, it can be envisaged that specific parts of the plant will not be depleted of N in the same way that shading or covering of individual plant sections can block photosynthesis and thus C supply. N starvation also results in a relative overabundance of C, and mitochondria may help “burn” this excess carbon [54]. In yeast, N starvation-induced mitophagy, but not general autophagy, was strongly inhibited in cells cultured on growth medium supplemented with lactate, a nonfermentable source of carbon requiring respiratory functions [55]. Therefore, mitophagy may strongly rely on the N/C interplay, and N and C starvation may produce opposing signals. In contrast, carbon starvation as a result of darkness triggers mitophagy within a few hours, indicating it may be part of the normal diurnal day/night cycle. During the night (or darkness in general) there is a major shift in ATP/energy production from the chloroplasts to the mitochondria, with mitochondrial respiration dominating as primary energy production process [56,57]. This increased workload for mitochondria thus likely results in an elevated need for mitochondrial quality control and turnover, which is thought to be one of the main functions of mitophagy. The carbohydrate (sugar) status of the cell affects starvation responses to control growth and also autophagy, with low sugar availability triggering increased autophagy [58]. The TOR kinase plays an important role here, as it inhibits autophagy under well-nourished conditions. During carbon starvation, TOR kinase is inhibited, thereby allowing autophagy to recycle cellular components to mobilize energy reserves. Our results show that inhibition of the TOR kinase is a very efficient inducer of mitophagy in plants. This thus suggests that the observed induction of mitophagy during extended darkness and carbon starvation is likely repressed by the TOR pathway under non-starvation conditions.

If the dark period is unusually long (e.g. longer than the standard 8 h during “long day” growth conditions), the rate of mitophagy increases dramatically (Figures 6A and 2B) and continues to increase as days in darkness go by. After prolonged dark periods, chloroplasts are among the first major components to be degraded, resulting in bleaching of the tissue (Figure 2A) [51]. Plant mitochondria are however retained until the last moments to support leaf metabolism and nutrient recycling [59]. Despite the strong drop in total protein content per mg fresh weight and strong increase in “mito-GFP” fusion protein processing (free GFP) as the dark period continues, the full-length mito-GFP protein levels stay relatively constant per mg tissue fresh weight (Figure 2B). Amino acids and lipids recycled during prolonged darkness by rapid induction of general autophagy and chlorophagy, can serve as substrates to support and sustain mitochondrial respiration for longer [60]. In other words, plant mitochondria are retained until the end and contain an increasingly large fraction of the total protein content as senescence progresses [51].

This further supports the idea that the main role of mitophagy is not to recycle mitochondrial components as energy reserves at the early stages of senescence, as may be the case for chlorophagy [61]. Plant mitochondria have indeed been shown to be present and active until the very last stages of (dark-induced) senescence, while chloroplasts are among the first cellular components to be degraded [51,62]. Moreover, plant mitochondria have been shown to become increasingly central energy and metabolic hubs to support cellular function and recycling as senescence progresses [59]. Thus, we propose that mitophagy is needed to maintain mitochondrial quality by removing damaged or dysfunctional individual mitochondria, to keep an overall healthy pool of mitochondria until the end. This is in agreement with the observation that a range of mitochondrial inhibitors that depolarize mitochondria (see also Ma et al. [32],) or interfere with mitochondrial biogenesis (either protein import or translation) are able to induce mitophagy. Somewhat surprisingly, we did not see a clear increase in mito-GFP turnover when the mitochondrial electron transport chain (mtETC) is inhibited by antimycin A. One explanation could be that the plant mtETC has remarkable flexibility and rapidly induces the alternative respiratory pathway via alternative oxidases and NADH dehydrogenases after AA treatment [63,64]. Although Dox, MB-6 and FCCP can also induce *AOX1a* expression [46], the alternative respiratory pathway would not be able to directly mitigate problems associated with uncoupling and biogenesis, as it would be in the case of cytochrome c mtETC inhibition by AA. Also in yeast [53] or mammals [65] AA on its own was shown not to be a strong trigger of selective mitophagy.

Besides dark-induced senescence/C starvation, also natural (age-related) senescence appears to be a major trigger for mitophagy in plants (Figure 6D-E). This is in further agreement with the observations made during dark-induced senescence, with both an overall relative increase in mitochondrial protein content as well as a relative increase in mito-GFP processing. Thus, also during natural senescence mitochondria likely need to remain functional and quality-controlled to assist with the final recycling of leaf components [51]. Mutants dysfunctional in autophagy display accelerated senescence phenotypes [31,40] (Figure S1C) and show significantly impaired mitophagy (Figure 5C). Therefore, it will be interesting to discover to what extent the lack of mitophagy contributes to this accelerated rate of senescence, but they are difficult to uncouple without very specific knowledge of the mechanisms behind mitophagy. Potentially, the lack of a well-maintained mitochondrial pool would impair the overall recycling efficiency and consequent “abandonment” of the leaf. Strikingly, *atg5-1* or *atg11-1* mutants, in which autophagic protein degradation is blocked, still exhibit a strong total protein degradation under prolonged dark conditions (Figure S2D), and although number of mitochondria in *atg* mutants was shown to be significantly stabilized under dark senescence, it still seemed to be lower as compared to the control light conditions [31]. It is therefore possible that other protein degradation or quality control mechanisms are accelerated in *atg* mutants to compensate for the autophagy and mitophagy loss. Some potential mechanisms might be the activation of organellar proteases like Clp [66], metalloprotease FtsH6 [67], or recruitment of the ABNORMAL SHOOT3 (ABS3) group transporters [68].

On the other end of the plant’s life cycle, mitophagy appears to be highly active in dark-germinated seedlings. Such a condition would occur in the natural environment when a seed is sown relatively deep or needs to evade an overlying obstacle. Also during etiolated growth, mitochondria are the main energy producers in the absence of developed chloroplasts. This again supports the notion that mitophagy is most active during times when the plant relies most strongly on mitochondria to provide energy or recycle nutrients. This may seem somewhat contradictory, as one could expect that it would be preferential to only remove mitochondria by autophagy after the period of high demand has passed, e.g. during the day. Conversely, the chance of getting most cellular damage by e.g. ROS production from damaged mitochondria would be highest during periods of peak mitochondrial activity. Therefore, it could be beneficial to remove the “low-quality” mitochondria during this peak usage time, to avoid unnecessary damage. The extended periods of etiolation in plants have been shown to promote the loss of seedling vigor and lower the rates of chlorophyll synthesis after transfer to white light. These effects were co-occurring with metabolic changes characteristic to carbon starvation [69]. In our system, *Arabidopsis* seedlings displayed moderate levels of general autophagy and mitophagy at the day 4–5 of darkness, after which chlorophyll levels upon transfer to WL were high, and the greening capacity was fully maintained (Figure S6B). However, when the etiolation periods were extremely long (8 days), the greening capacity upon transfer to WL was strongly impaired, and this phenotype correlated with the higher rates of mitophagy and general autophagy processing (Figure S6B). This may be because the seedling is severely depleted of energy reserves after such a long time in the dark and has survived the starvation by degrading cellular components needed for efficient photomorphogenesis upon light exposure.

Remarkably, mitophagy was completely suppressed in the de-etiolated seedlings after as little as 6 h of light exposure (Figure 7B). This is in contrast to a previous study that reported an increase in mitophagy, as observed by electron microscopy and a reduction in overall mitochondrial protein content, specifically at 8 h after transfer to light of etiolated seedlings [32]. We also collected samples at 8 h post de-etiolation, but we could only observe that the high level of mitophagic flux in the etiolated seedling was nearly completely lost after 8 h in the light. This possible discrepancy may be because of the potential difficulty of accurately quantifying mitophagy across whole tissues using electron microscopy, as opposed to using GFP-based flux assays and confocal microscopy. Our findings indicate that mitophagy can be very tightly controlled by as yet unknown mechanisms. It also implies that the observed increase in free GFP obtained in our immunoblots is likely representing a continuous turnover of mitochondrial components by mitophagy, rather than an accumulation of unprocessed GFP over time. In that sense our method is likely really measuring mitophagic flux. As mitochondrial protein content does not seem to decrease strongly despite the increased rates of mitophagy e.g. during dark-induced senescence (Figure 2B), it seems probable that mitochondrial turnover by mitophagy is balanced by continuous mitochondrial biogenesis to maintain a sufficiently large pool of active mitochondria.

In conclusion, this work provides a significant increase in our understanding of the conditions that induce mitophagy in plants, which to some extent appear to be different than in other organisms and not closely linked to general stresses. In particular, mitophagy appears to be at its most active during periods of low photosynthetic activity such as dark germination and (extended) nighttime, or during dark/age-induced senescence. Likely, mitophagy operates to keep a healthy mitochondrial pool during these periods of increased mitochondrial demand. Reduced mitochondrial capacity to maintain membrane polarization or protein import/translation may be part of the signal that tags specific dysfunctional mitochondria for mitophagic degradation. Furthermore, the newly-developed reporter system presented here will be a useful tool to study mitophagy in future research.

## Materials and methods

### Plant materials and growth conditions

All *Arabidopsis thaliana* lines used in this study, including the wild type, originate from the same Columbia-0 (Col-0) ecotype. Two autophagy mutant lines: *atg5-1* (SAIL_129_B07; [40]) and *atg11-1* (SAIL_1166_G10; [31]) were obtained from the Nottingham Arabidopsis Stock Centre (NASC), and characterized in this study (Figure S1). Plants homozygous for the T-DNA insertion were confirmed by genotyping, using primers listed in Table S1.

Unless specified otherwise, for all in vitro plant growth conditions, seeds were surface sterilized with 70% (v:v) ethanol for 5 min and 20% (v:v) commercial bleach (Klorin brand, Colgate; contains 2.7 g/L sodium hypochlorite) for 10 min, washed 6 times with sterile water, then sown on half-strength Murashige and Skoog (MS) medium (Duchefa, M0222) supplemented with 1% (w:v) sucrose (Duchefa, S0809), 0.05% (w:v) 4-morpholineethanesulfonic acid (MES; Biomol, 06010) and 0.8% (w:v) agar (Duchefa, P1001) (pH 5.7). Seeds were imbibed for 3 d at 4°C in darkness, and seedlings were grown under the photoperiodic cycle of 16 h light (WL, 80–100 μmol m^−2^ sec^−1^) at 22°C and 8 h dark at 19°C, with relative humidity at 60%. For selection of transgenic lines, growth medium was additionally supplemented with 10 µg ml^−1^ Basta (Alfa Aesar, J66186), or 40 µg ml^−1^ hygromycin (Cayman Chemical, 14,291). For etiolation assays, seedlings were sown on half-strength Murashige and Skoog (MS) medium, without sucrose, with 0.8% (w:v) agar (pH 5.7). Following 3 d stratification in the dark at 4°C plates were incubated in WL for 2 h to induce germination, covered in 2 layers of aluminum foils and left in the dark room to continue growth for 4–6 d. To induce de-etiolation, foil was removed and plates were transferred to standard light conditions for 6–24 h. For the age-dependent senescence (natural senescence) and hypoxia assays, seeds were sown on soil mix consisting of soil, perlite and vermiculite (in a 4:1:1 ratio), stratified for 3 d at 4°C in darkness and left to grow under the standard long day growth conditions, as specified above.

### Transgenic plants generation

To produce mitochondrial IDH1, TOM20-2 and TOM20-3 GFP fusion lines, full coding regions (including the stop codon for the GFP-TOM20-2 and GFP-TOM20-3 N-term fusion, but excluding it for the IDH1-GFP C-term fusion) were amplified from cDNA. cDNA was synthesized from the Col-0 using an iScript cDNA Synthesis Kit (Bio-Rad, 1,708,891), according to the manufacturer’s instructions. The amplified PCR products were cloned into the Gateway entry vector *pDONR^TM^* 221 using BP Clonase II (Thermo Fisher Scientific, 11,789,020), and positive sequence-verified entry vectors were recombined into destination vectors: pH7WGF2 (TOM20-2 and TOM20-3 cloning) or pB7FWG2 (IDH1 cloning) by LR Clonase II (Thermo Fisher Scientific, 11 791 020). The construct to produce general autophagy fluorescent marker line 35S::GFP-ATG8e was generated by the same Gateway strategy as described above (Invitrogen, CA, USA), while full length coding sequence of *ATG8e* was amplified from the Col-0 cDNA, and recombined into pB7WGF2 destination vector by LR clonase II. Second general autophagy marker line, 35S::GFP-ATG8a, was kindly gifted by Prof. Richard Vierstra (Washington University in St. Louis). To generate the *35S::CoxIV-mCherry* construct, the mitochondrial *CoxIV* targeting sequence corresponding to the first 29 aa of the yeast (*Saccharomyces cerevisiae*) COX4 (cytochrome c oxidase subunit 4) [70], and the *mCherry* sequence (derived from the plasmid described in [71]), were amplified using primers, that introduce BamHI restriction sides at the 3ʹend of the *CoxIV* and 5ʹend of the *mCherry* (Table S1). The amplified PCR products were first digested with the BamHI, ligated with the T4 Ligase (Thermo Fisher Scientific, EL0014), cloned into *pDONR^TM^* 221 entry vector, and finally recombined into 35S expression vector pH7WG2 following LR reaction. Wild type, *atg5-1, atg11-1*, or *35S::GFP-ATG8a* plants were transformed with the *Agrobacterium tumefaciens* (strain GV3101) by the floral dip method [72]. Details on all primers used for cloning are listed in Table S1.

### Plant stress treatments

## Starvation treatments

For nitrogen starvation, seedlings were grown on half-strength MS medium with 1% sucrose, 0.05% MES and 0.8% agar for 10 d under standard photoperiodic conditions. Whole seedlings were gently transferred, with tweezers, to the same fresh media (mock) or to half-strength MS medium without nitrogen (Merck, M0529) with 1% sucrose, 0.05% MES and 0.8% agar. Plants were returned to the normal growth conditions for an additional 1, 3 and 6 days.

For carbon starvation/dark senescence, 10 d old seedlings grown on half-strength MS medium without sucrose and with 0.8% agar were wrapped in double aluminum foil and grown under the same standard growth conditions as before for another 1 up to 8 days.

## Natural senescence

The age-dependent senescence experiment was performed as described in [73], with minor modifications. *35S::GFP-TOM20-3* and *35S::GFP-ATG8e* plants were sown onto soil and grown under optimal, long photoperiodic conditions. 10 d after germination, the first true leaves emerged, and since then, the leaf age count begun. For each western blot sample, whole third and fourth rosette leaves were cut at the bottom of the petiole with sharp tweezers.

## Abiotic stresses

For the UV-B and H_2_O_2_ stresses seeds were germinated for 10 days on half-strength MS medium, supplemented with 1% sucrose, 0.05% MES and 0.8% agar. The UV-B stress was applied by transferring agar plates, without the cover lids into a UV-B XL-1000 Crosslinker (Spectrolinker™, Spectronics Co., New York, NY, USA) equipped with 4 lamps (type BLE-8T254, SW, 8 W each, Spectronics Co.). Seedlings were exposed to UV at a dose of 10,000 mJ cm^−2^. Immediately after, plates were covered and returned into the normal growth condition, for recovery in standard growth conditions through 8–48 h.

The H_2_O_2_ stress was applied by spraying 100 mM H_2_O_2_ (Merck, 95,321) solution in water to the agar grown seedlings, from a short distance using plastic spray bottle. The control mock samples received the same amount of sprays of just MQ water. Seedlings were left in the growth chamber for the next 8–48 h or 48 h only (mock) and sampled at the indicated times.

Heat thermopriming assay was applied from [74], with minor modifications. In general, 7 d old seedlings were exposed to mild heat tress at 37°C for 1.5 h in the dark, then returned to 22°C for another 1.5 h, followed by 50 min of severe heat stress at 44°C. Mild heat was applied by transferring plates into 37°C incubator, while for the heat stress at 44°C, Petri dishes covered with the aluminum foil were immersed in the hot water bath. Seedlings were returned to the normal photoperiodic growth conditions for a thermorecovery faze at 22°C for up to 3 d. Tissue was collected and frozen in liquid nitrogen at the indicated times.

Hypoxia experiment was adapted from [45], where 3 to 4 whole rosette leaves from 5-week-old, soil grown plants, were cut at the base and submerged in assay medium on 24-well transparent plates. Following a short 3 min vacuum infiltration wells were topped up with the same assay medium and sealed with the transparent film (Bio-Rad, MSB1001) to induce hypoxia. Control samples were left open to allow gas exchange. Plates were incubated under constant low WL (10 μmol m^−2^ sec^−1^) for 24 h before sampling. Alternatively, 12-d-old *Arabidopsis* seedlings grown on a 9 mm Petri dishes, on 1/2 MS agar medium with 1% sucrose (pH 5.7) and 5 mM MES, were placed into the anaerobic jar (Oxoid) supplemented with the AnaeroGen™ 2.5 L sachets (Thermo Fisher Scientific, AN0025A). Sealed jars were left in darkness for 24 h before sampling. The AnaeroGen reduces oxygen level in the jar below 1% within 30 min, simultaneously generating 9–13% CO_2_.

## Treatments with mitochondrial chemical inhibitors

Antimycin A (AA), doxycycline (Dox), MitoBlockCK-6 (MB) and carbonyl cyanide-4 (trifluoromethoxy) phenylhydrazone (FCCP; Merck, C2920) spray treatments from Figure 4A-C were performed as described before [46]. In brief, 50 μM AA (Merck, A8674), 25 μg/ml Dox (Merck, D9891), 50 μM MB (Tebu-bio, 10–1472), or 20 μM FCCP (Merck, C2920) dissolved in water (with an additional 0.01% Tween-20 [Merck, PP1379]), were sprayed to various 10-d-old transgenic lines grown on half-strength MS medium with 1% sucrose, 0.05% MES and 0.8% agar. Plants were left in the standard photoperiod conditions for up to 24 h. Samples were collected at various time points indicated in the figure legend.

Longer exposure to mitochondrial inhibitors was performed by manually transferring 10 d old seedlings from the standard agar growth media to the new agar medium supplemented with 30 μM AA, 15 μg/mL Dox, 20 μM MB or 10 μM FCCP. Plants were left to continue growth under photoperiodic light for the next 2, 4 and 6 d or collected immediately before the transfer. For the mock controls seedlings were transferred to a fresh growth medium without any inhibitors.

## Treatments with chemical autophagy inhibitors

To treat plants with TOR kinase inhibitor (AZD8055; Fisher Scientific, 17,187,109), phosphatidylinositol-3-kinase inhibitor (wortmannin; Fisher Scientific, 15,406,129), or vacuolar degradation inhibitors: concanamycin A (ConA; Merck, 27,689) or E-64d (Cayman Chemical, VWR, CAYM13533), with or without additional proteases inhibitor (pepstatin A; Fisher Scientific, 15473039), 7 d old seedlings grown vertically were transferred to liquid 1/2 MS medium containing 10 μM AZD8055, 5 μM wortmannin, 0.5–1 μM ConA, or 20 μM E-64d, with or without 10 μM pepstatin A, or an equal volume of DMSO (0.1% [v:v]; VWR, 0231) as a control for 16 h up to 2 days (depending on the assay), before subsequent microscopy, or sample collection for the immunodetection. For all experiments requiring dark treatment seedlings were kept in the dark room, covered with aluminum foil. If samples were subjected for imaging, microscopy slides were prepared in the dark under a dim green safelight. For the double mitochondria inhibitors treatment with ConA or abiotic stresses with ConA, samples were left under constant while light before imaging, unless not specified otherwise.

### Oxygen consumption quantification for multiwell plate-based hypoxia assay

The Extracellular Oxygen Consumption Assay Kit (Abcam, ab197243) was used to monitor changes in oxygen concentration in a transparent 96-well plate with the *Arabidopsis* wild type (Col-0) leaf pieces (30 mg per single well) cut from 5-week-old plants. The fluorescent dye was reconstituted in water according to the manufacturer’s instructions and added to individual wells with or without leaf tissue submerged in assay medium in a 1:15 ratio of dye:medium. Control samples were left opened, while samples subjected to the hypoxic condition were sealed with an oil supplemented with the kit. Fluoresce signal intensities of the dye were measured with excitation (Ex) and emission (Em) set at Ex = 380 ± 10 nm/Em = 650 ± 10 nm. Fluorescence signals were recorded for 24 h, every 10 min at 25°C using a BMG CLARIOstar plate reader (Labtech, Germany).

### qRT-PCR analysis of hypoxia stress marker gene expression

Total RNA was extracted from a frozen wild type (Col-0) seedlings exposed to hypoxic conditions as described above using the Spectrum^TM^ Plant Total RNA Kit (Sigma Aldrich, STRN250) according to the manufacturer’s instructions. Possible genomic DNA contaminations were removed during the RNA extraction by introducing an additional on-column DNase I treatment step (Sigma Aldrich, DNASE70-1SET), after RNA binding to the column, following the manufacturer’s instructions. 600 ng of RNA was reverse transcribed in one step using iScript cDNA Synthesis Kit (Bio-Rad, 1708891) according to the manufacturer’s instructions. Transcripts abundance was quantified by real-time PCR (qRT-PCR) reaction using SsoAdvanced^TM^ Universal SYBR Green Supermix (Bio-Rad, 1725271). Each reaction content and thermal cycling conditions were as described before [46]. Sequences for gene-specific primers and accession numbers used for qRT-PCR are listed in Table S1.

### Protein extraction and western blot analysis

Total cellular protein was extracted from whole Arabidopsis seedlings, or cotyledons with hypocotyls tissue for the dark etiolation assays. Plant tissues were ground using a TissueLyser (Retsch MM300, Qiagen, Hilden, Germany) to a fine powder in liquid nitrogen. Each 100 mg tissue was homogenized in 500 µL 2x Laemmli sample buffer (0.125 M Tris-HCl, pH 6.8, 4% [w:v] SDS, 20% (v:v) glycerol [VWR, 24388.295]), containing 5% (v:v) 2-mercaptoethanol (Merck, 63689), and cOmplete Protease Inhibitor Cocktail (Roche, 11873580001), added freshly to a 1x final concentration. Samples were incubated on ice for 10 min, denatured at 98°C for 5 min, and cleared by centrifugation at 16,000 x *g* for 5 min at 4°C.

Resulting protein extracts were separated by SDS/PAGE using precast 4–20% Mini-PROTEAN TGX Protein Gels (Bio-Rad, 4,568,096). Equal volume of the total protein extracts was loaded per each well and each sample. Proteins were blotted to 0.2 µm PVDF membrane (Bio-Rad, 1,704,156), using a semi-dry Turbo transfer system (Bio-Rad). Membrane blocking was performed in 5% (w:v) skimmed milk in TBS (50 mM Tris-Cl, pH 7.6, 150 mM NaCl) for 1 h, which was followed by an incubation with primary anti-GFP antibody (Thermo Fisher Scientific, A-6455) overnight at 4°C, using 1:5000 dilution in 5% skimmed milk in TBST. Membranes were washed 6 times, 5 min each, with TBS-T. For the detection, an anti-rabbit horseradish peroxidase (HPR)-conjugated antibody (Merck, A9169) at 1:10,000 dilution was used. The immunoreaction was developed using Clarity Western ECL Substrate (Bio-Rad, 1705060) and was detected in a ChemiDoc XRS system (Bio-Rad). All experiments were repeated with at least three biological repeats and representative results are shown in the figures.

### Chlorophyll measurements

Chlorophyll measurements were performed for 20 seedlings grown in the dark for 4, 6 or 8 d and transferred to constant WL for 24 h. Seedlings were ground to a powder in liquid nitrogen and incubated on ice for 3 min in 800 µL of 80% cold acetone, with occasional vortexing. After centrifugation at 16,000 x *g* for 5 min at 4°C, supernatants were subjected to spectrophotometric measurements. Absorbance values at 647 and 663 nm were recorded and total chlorophyll levels calculated according to [75].

### Confocal microscopy analysis

Confocal laser scanning microscope, Leica TCS-SP8 was used to acquire cell fluorescence images, with the 25x water objective lens. For the root tissue, imaging was performed in the root differentiation zone, and for the cotyledons, in the epidermal cells from the abaxial side. Excitation/detection parameters for GFP, mCherry and chlorophyll auto-fluorescence were 488 nm/490-550 nm, 561 nm/560-630 nm, 640 nm/660-700 nm, respectively. For the multiple fluorophore colocalization imaging, the sequential scanning mode was used, to avoid signal bleed-through. All confocal data was processed with Fiji and ImageJ (version 1.52a) software.

## Supplementary Material

Supplemental Material

## Statistical analysis

All the quantification data was presented as mean ± SE and includes statistical analysis. Statistical significance between two experimental groups was analyzed by a two-tailed Student’s *t*-test. Differences between individual groups were considered significant if *p* < 0.05 (*); *p* < 0.01 (**).
