# Supplementary material for: Carbon starvation, senescence and specific mitochondrial stresses, but not nitrogen starvation and general stresses, are major triggers for mitophagy in Arabidopsis
Source: Autophagy. 2022 Apr 3;18(12):2894–912. doi: 10.1080/15548627.2022.2054039 (PMC9673927; doi:10.1080/15548627.2022.2054039)
Supplement: Supplemental Material [file KAUP_A_2054039_SM5157.docx]

**Carbon starvation, senescence and specific mitochondrial stresses, but not nitrogen starvation and general stresses, are major triggers for mitophagy in Arabidopsis.**

Sylwia M. Kacprzak, Olivier Van Aken*

Department of Biology, Lund University, Lund, Sweden

*Corresponding author: Olivier Van Aken

Molecular Cell Biology

Department of Biology

Lund University

Sölvegatan 35

Lund 223 62 – Lund, Sweden

Tel: +46 76 210 14 03

e-mail: olivier.van_aken@biol.lu.se

ORCID: Olivier Van Aken (0000-0003-4024-968X); Sylwia M. Kacprzak

**Supplementary Information**

**
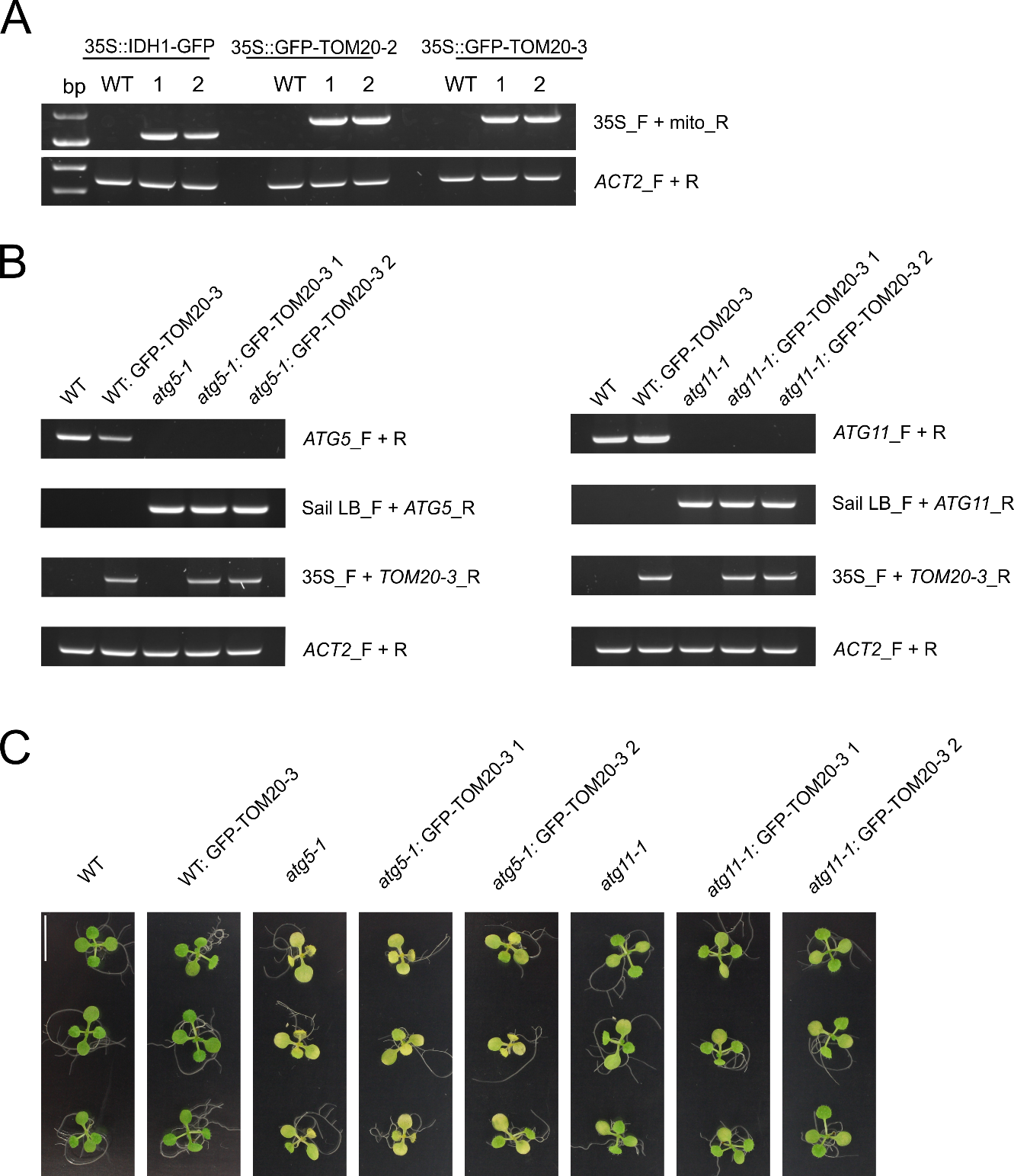
**

**Figure S1.** Genotyping and characterization of WT *35S::IDH1-GFP*, *35S::GFP-TOM20-2* and *35S::GFP-TOM20-3,* as well *atg5-1* *35S::GFP-TOM20-3* and *atg11-1* *35S::GFP-TOM20-3* transgenic lines. (**A**) PCR analysis of transgenic cassette insertion. Genomic DNA from Col-0 and various transgenic lines was amplified using 35S promoter specific primer (35S_F) and mitochondrial gene specific primers (collectively named mito_R). Primers binding ACTIN2 (ACT2_F+R) were used as a positive control. (**B**) PCR analysis of SAIL T-DNA, and *35S::GFP-TOM20-3* transgenic cassette insertions. Genomic DNA from WT (Col-0), *atg5-1* and *atg11-1* seedlings was amplified using *35S* promoter and *TOM20-3* gene specific primers (35S_F, TOM20-3_R), *ATG5,* or *ATG11* genes specific primers (ATG5/11 F+R), and left border specific primer (Sail Lb + ATG5/11_R). *ACTIN2* transcript was amplified as a control. (**C**) Photographs of the de-greening phenotypes induced by carbon starvation in WT (Col-0), *atg5-1* and *atg11-1* seedlings, stably transformed with or without the *35S::GFP-TOM20-3* construct. 10-d-old *Arabidopsis* seedlings grown on 1/2 MS medium without sucrose were transferred to constant darkness for 6 days. Scale bar: 7 mm.

**
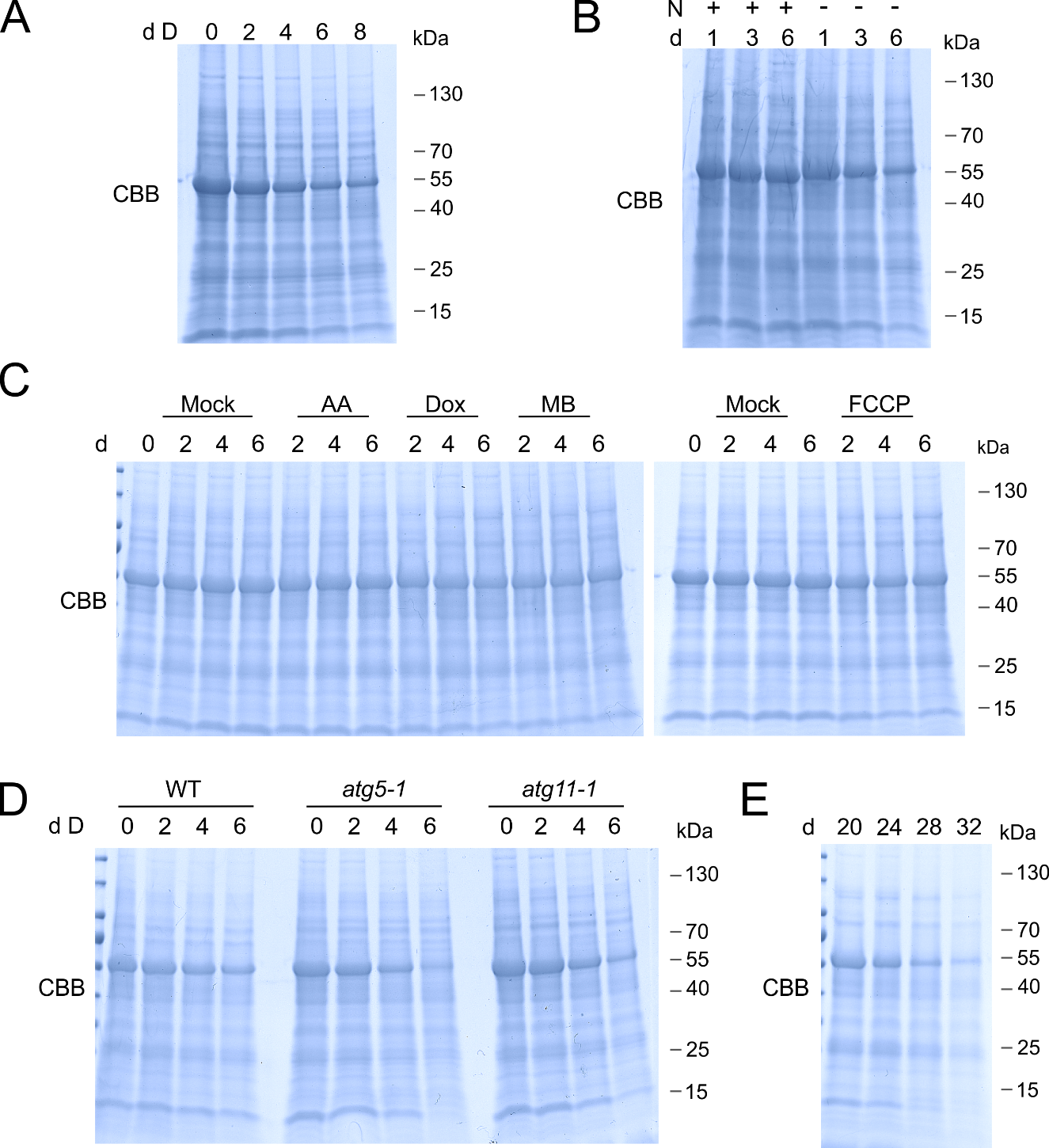
**

**Figure S2.** Coomassie Brilliant Blue (CBB) staining of representative total cellular protein extracts resolved on SDS-PAGE, from *35S::GFP-TOM20-3* transgenic plants, treated with various stresses, and subjected to immunoblot analysis in this work. (**A**) 10-d-old WT *35S::GFP-TOM20-3* seedlings subjected to carbon starvation (dark induced senescence) stress presented in Figure 2A-B. (**B**) 10-d-old WT *35S::GFP-TOM20-3* seedlings grown on media without N, shown in Figure 2E-F. (**C**) 10-d-old WT *35S::GFP-TOM20-3* seedlings transferred to 1/2 MS agar medium supplemented with various mitochondrial chemical inhibitors, shown in Figure 4D-E. (**D**) Seedlings of WT, *atg5-1* and *atg11-1* *35S::GFP-TOM20-3* subjected to carbon starvation stress shown in Figure 5C. (**E**) Rosette stage WT *35S::GFP-TOM20-3* plants undergoing natural senescence presented in Figure 6C-E.

**
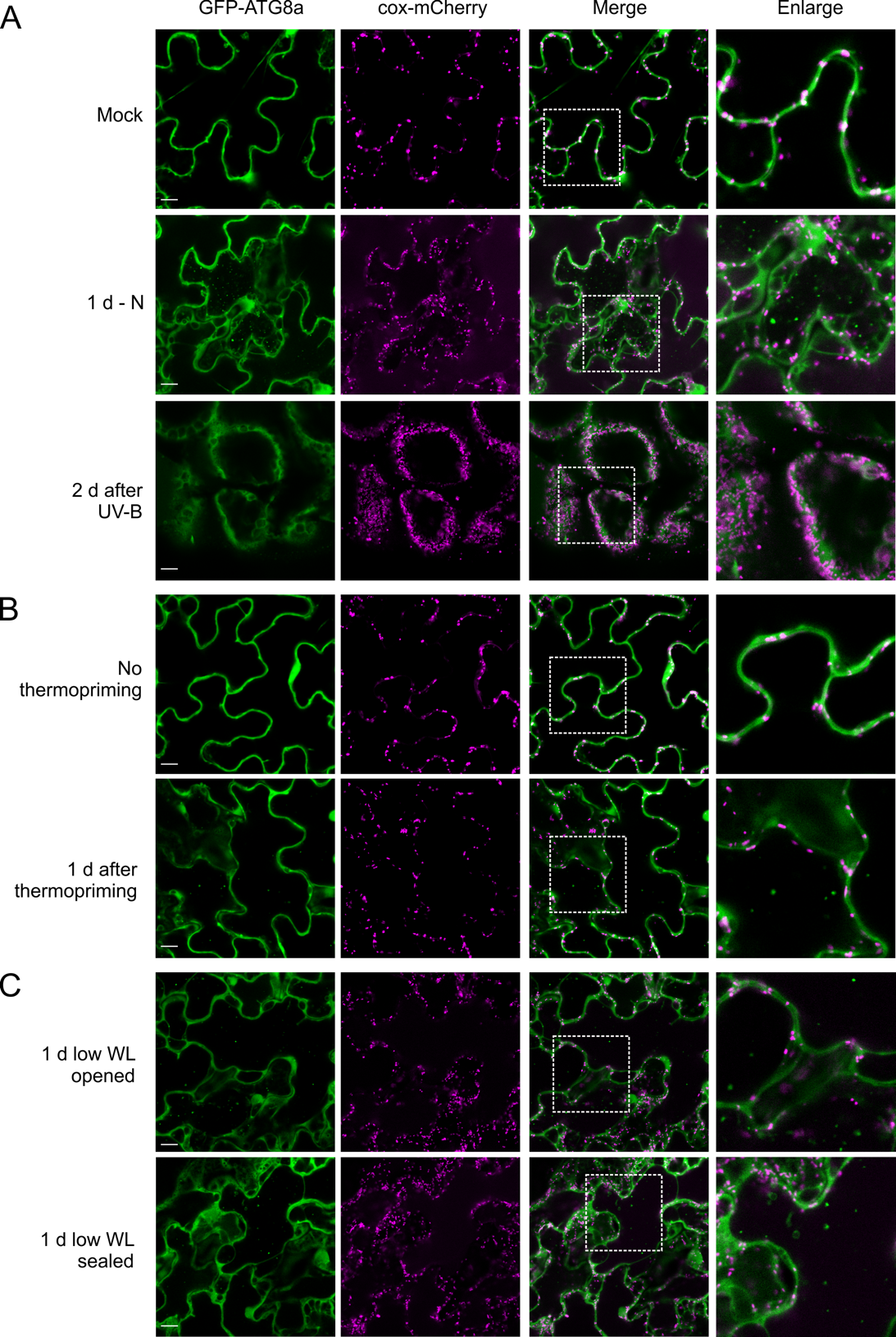
**

**Figure S3. Fluorescence confocal microscopy** analysis of mitochondria and autophagosome localization during various stresses. Fluorescence confocal microscopy analysis of colocalization of mitochondria with GFP-ATG8a autophagosomes in cotyledons of 7-d-old seedlings expressing 35S::GFP-ATG8a and 35S::cox-mCherry. Scale bars: 10 µm. (**A**) For UV-B stress, 5-d-old seedlings were exposed to 10 000 mJ cm^-2^ UV-B and returned to standard growth conditions for recovery for 2 d. 24 h before imaging, seedlings were incubated under constant WL in the liquid 1/2 MS media with 1% sucrose supplemented with 1 μM concanamycin A (ConA) (Mock, UV-B) or the same medium without N source (Merck; M0529) to induce N starvation stress. (**B**) For heat stress recovery, 5-d-old seedlings grown on 1/2 MS agar plates with 1% sucrose were thermoprimed as described in Figure 3C and returned to standard growth condition for 2 d. Four h before imaging, heat primed and non-primed (mock) seedlings were incubated in the dark in the liquid 1/2 MS media supplemented with 1 μM ConA. (**C**) For hypoxia stress, 6-d-old seedlings were incubated for 24 h under low WL in a 96-well plate in assay medium supplemented with 1 μM ConA. Control samples were left open, while treated samples were sealed with the transparent film to block O_2_.

**
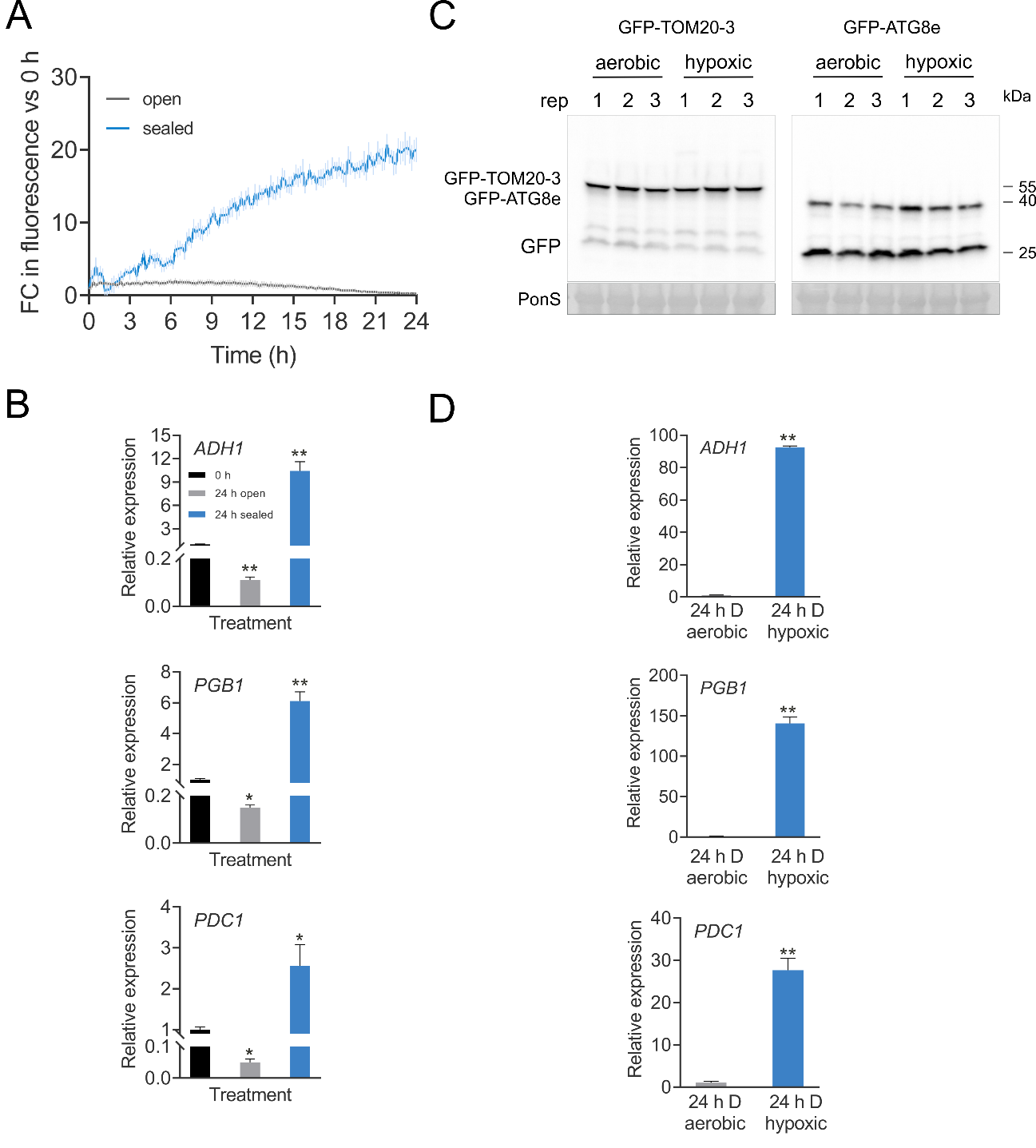
**

**Figure S4.** Confirmation of the low oxygen stress induction in a multiwell plate set up, and additional evaluation of mitophagy induction by hypoxic stress treatment in the dark using AnaeroGen system. (**A**) Evaluation of the oxygen (O_2_) consumption in wells using Extracellular Oxygen Consumption reagent. Wells containing tissue from Col-0 rosette leaves submerged in the assay medium with the dye were either left open (gray) or sealed from O_2_ with the oil supplemented with the kit (blue). The dye fluorescence is quenched by O_2_, therefore O_2_ consumption results in an increase of the dye fluorescence signal. Data points represent mean (± SE) of fold-change (FC) in relative florescence signals vs the first measurement, at the time point 0 h, for a given treatment (*n*=3). Fluorescence of the dye in assay medium, without plant tissue, was additionally recorded (blank) and subtracted from the florescence signals from samples containing plant material. (**B**) Col-0 seedlings were submerged in assay medium in 96-well plate (30-40 mg tissue per well) and sealed with the transparent film to induce hypoxic conditions or left open, and maintained 24 h under low WL as in Figure 3. Hypoxia stress marker gene expression was determined by qRT-PCR and is relative to untreated Col-0 before submergence (0h)(n=3). Asterisks denote a significant difference vs. 0 h, Student's t-test (*p<0.05, **p<0.01). ADH1, ALCOHOL DEHYDROGENASE1; *PGB1*, PHYTOGLOBIN1; *PDC1*, PYRUVATE DECARBOXYLASE1; (**C**) GFP-TOM20-3, GFP-ATG8e fusion proteins, and free GFP were detected by immunoblotting using an anti-GFP antibody, after 24 h hypoxia stress in the dark generated by placing plate grown 12-d-old seedlings into jars with AnaeroGen sachets. (**D**) Analysis of hypoxia marker gene egression by qRT-PCR for the Col-0 seedlings treated as in (C). Asterisks denote a significant difference vs. plate grown seedlings kept in darkness air open (24 h D aerobic), Student's t-test (**p<0.01).


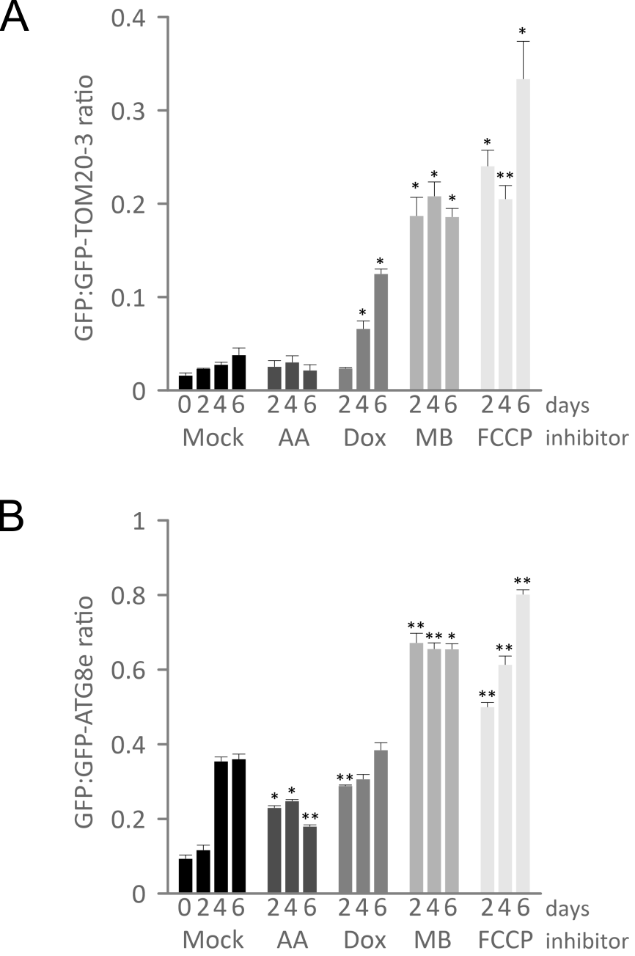


**Figure S5.** Ratios of free GFP to full-length GFP fusion proteins calculated from immunoblots presented in Figure 4E. Bars shown are the means (± SE) of three biological replicates (Student’s *t*-test; *p < 0.05, **p < 0.01). Asterisks denote a significant difference for GFP-TOM20-3 (**A**), or GFP-ATG8e (**B**) for a given mitochondrial inhibitors (AA, Dox, MB or FCCP) versus the Mock treated samples for the same number of days (2, 4 or 6) after inhibitor transfer.

**
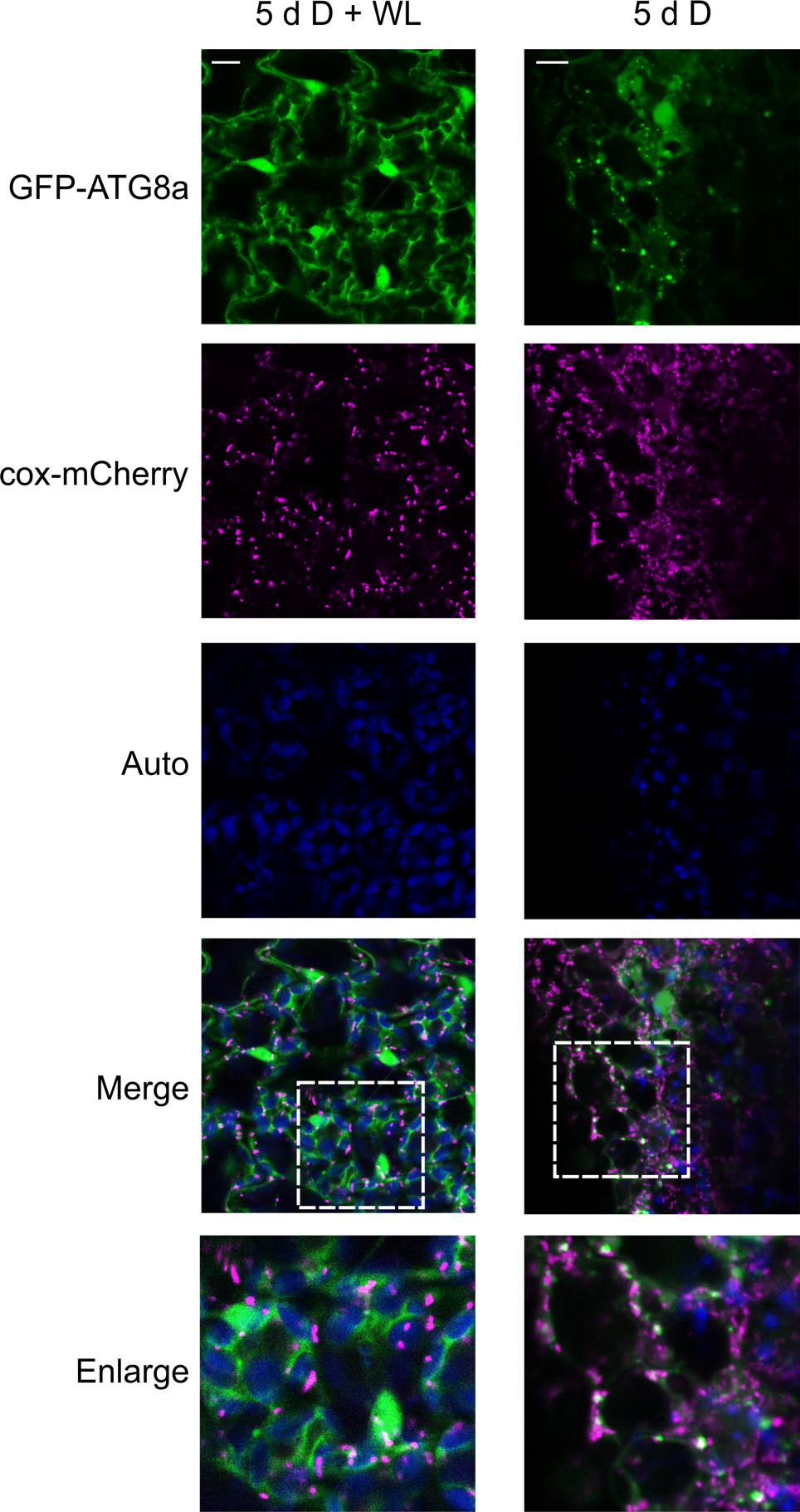
**

**Figure S6.** Confocal microscopy images of cotyledons from etiolated seedlings grown in darkness for 5 days, or followed by an additional white light exposure for 24 h. Colocalization of mitochondria with GFP-ATG8a autophagosomes in cells of abaxial cotyledon epidermis from etiolated transgenic line expressing *35S::GFP-ATG8a* and *35S::cox-mCherry*, by confocal fluorescence microscopy. **Twenty-four h before imaging, seedlings were incubated in liquid 1/2 MS media** supplemented with 0.5 μM concanamycin A (ConA) under constant WL (left panel) or in the dark (right panel)**.** Scale bars: 10 µm.

**
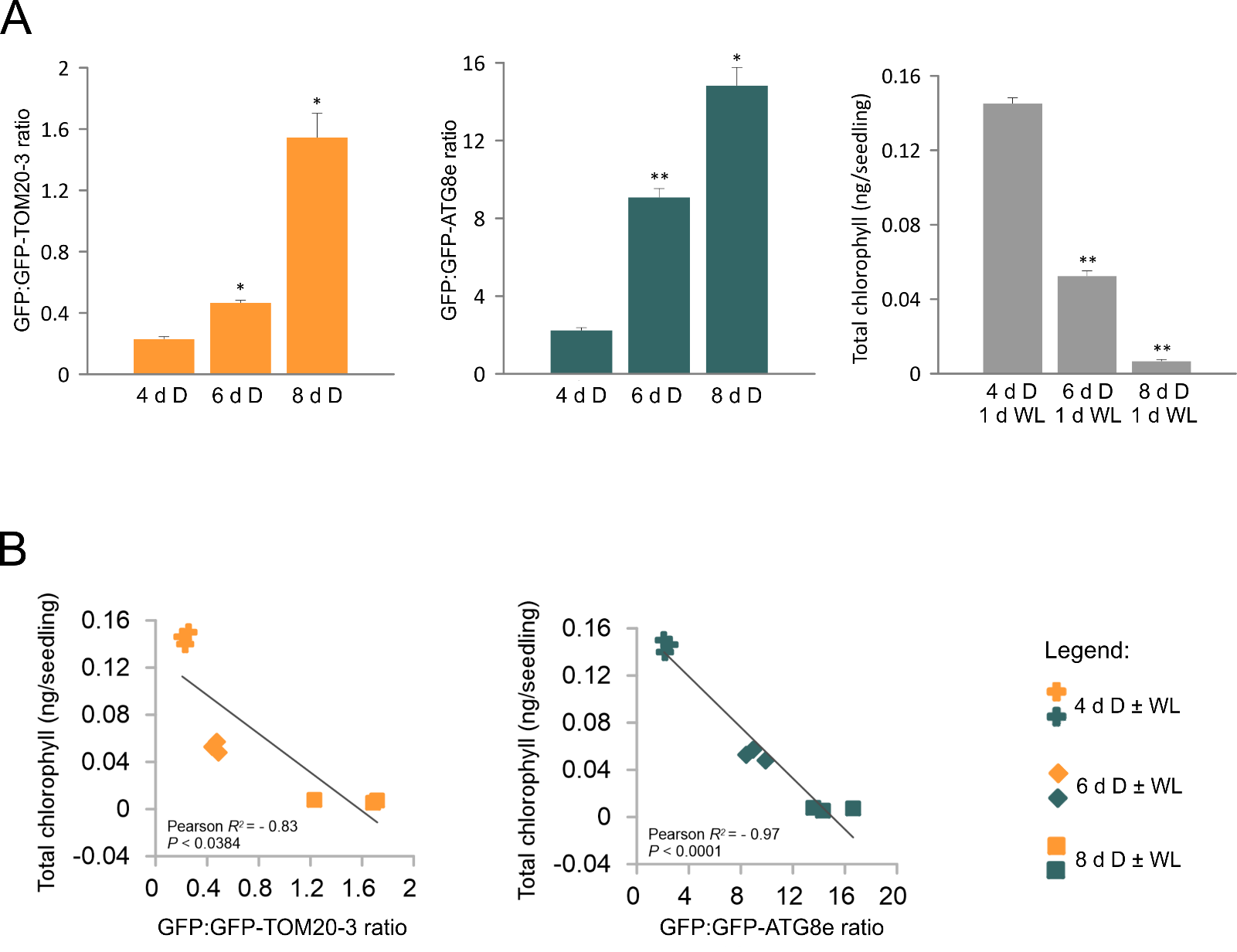
**

**Figure S7.** Correlation of mitophagy and general autophagy during etiolation with greening capacity. (**A**) Vertical bar charts illustrating ratios of free GFP to GFP-TOM20-3 or GFP-ATG8e of immunoblots in Figure 7A, from etiolated seedlings (left and middle panel), or accumulation of total chlorophylls in WT (Col-0) de-etiolating seedlings after exposure to white light (WL)(right panel). Bars shown are the means (± SE) of three biological replicates (Student’s *t*-test; *p < 0.05, **p < 0.01). Asterisks denote a significant difference versus signal ratios (or total chlorophyll accumulation) at the day 4 (±WL). (**B**) Correlation plots between changes in free GFP to GFP-fusion protein ratios during dark germination and total chlorophyll levels in etiolated seedlings after transfer to WL for 24 h. Data points represent mean chlorophyll levels (n=20 seedlings) plotted against individual protein ratio (n=3) for each transgenic line.

**Table S1.** Primers used for molecular cloning of various genes described in this work, genotyping of *Arabidopsis* transgenic plants, or autophagy mutants and analysis of hypoxia stress marker gene expression

| **Primer Name** | **Gene name & AGI** | **Primer sequences (5’ > 3’)** | **Purpose** |
| --- | --- | --- | --- |
| IDH1_GW_F | *IDH1*  AT4G35260 | GTACAAAAAAGCAGGCT**TC**ATGTCTCGCAGATCGCTAA | Amplification of the *IDH1* coding sequence without a stop codon, to add partial Gateway® *attB* recombination sites at each end (1^st^ step of a two-step PCR reaction) |
| IDH1_GW_R |  | TACAAGAAAGCTGGGT**G**GTCTAGTTTTGCAATGACCG |  |
| TOM20-2_GW_F | *TOM20-2*  AT1G27390 | GTACAAAAAAGCAGGCT**TC**ATGGAGTTCTCTACCGCC | Amplification of the full-length *TOM20-2* coding sequence to add partial Gateway® *attB* recombination sites at each end (1^st^ step of a two-step PCR reaction) |
| TOM20-2_GW_R |  | TACAAGAAAGCTGGGT**G**TTATCTGGCAGGAGGTGGA |  |
| TOM20-3_GW_F | *TOM20-3*  AT3G27080 | GTACAAAAAAGCAGGCT**TC**ATGGATACGGAAACTGAGT | Amplification of the full-length *TOM20-3* coding sequence to add partial Gateway® *attB* recombination sites at each end (1^st^ step of a two-step PCR reaction) |
| TOM20-3_GW_R |  | TACAAGAAAGCTGGGT**G**TTAACGAGGAGGAGAGACA |  |
| ATG8e_GW_F | *ATG8e*  AT2G45170 | GTACAAAAAAGCAGGCT**TC**ATGAATAAAGGAAGCATCTT | Amplification of the full-length *ATG8e* coding sequence to add partial Gateway® *attB* recombination sites at each end (1^st^ step of a two-step PCR reaction) |
| ATG8e _GW_R |  | TACAAGAAAGCTGGGT**G**TTAGATTGAAGAAGCACCGA |  |
| mCherry_Bam_F | *mCherry*  *-* | GCAGGATCCATGGTGAGCAAGGGCGAG | Amplification of the full-length *mCherry* coding sequence to add the BamHI restriction site at 5’ end, and the full Gateway® *attB2* recombination site at 3’ end |
| mCherry_GW_R |  | GGGGACCACTTTGTACAAGAAAGCTGGGT**G**TTACTTGTACAGCTCGTCCAT |  |
| CoxIV_GW_F | *CoxIV*  - | GGGGACAAGTTTGTACAAAAAAGCAGGCT**TC**ATGCTTTCACTACGTCAATCTATAA | Amplification of the *CoxIV* targeting peptide coding sequence to add the full Gateway® *attB1* recombination site at 5’, and the BamHI restriction site at 3’ end |
| CoxIV_Bam_R |  | GCAGGATCCGGGTTTTTGCTGAAGCAGATATC |  |
| attB1 | *-* | GGGGACAAGTTTGTACAAAAAAGCAGGCT | Addition of the full length attB1/2 Gateway® recombination sites at each end (2^nd^ step of a two-step PCR reaction) |
| attB2 | *-* | GGGGACCACTTTGTACAAGAAAGCTGGGT |  |
| ATG5-1_F | *ATG5*  AT5G17290 | ATTTGCTATTTGTTTGGCACG | Primers specific to *ATG5* coding sequence for PCR genotyping *atg5-1* mutant (wild type band) |
| ATG5-1_R |  | TACCGTTCATGACAGAGGTCC |  |
| ATG11-1_F | *ATG11*  AT4G30790 | TGCATTTGTTTTTCCTTGAGC | Primers specific to *ATG11* coding sequence for PCR genotyping *atg11-1* mutant (wild type band) |
| ATG11-1_R |  | AAGTGTCCTCATCTCTGCGTC |  |
| SAIL_LB | - | GTGTACCAAACAACGCTTTACAGC | T-DNA primer for PCR genotyping *atg5-1* and *atg11-1* mutants (mutant band) |
| 35S_F | - | GAGGACTCCGGTATTTTTACAAC | *35S* promoter primer for PCR genotyping of transgenic lines |
| IDH1_R | *IDH1*  AT4G35260 | CCGGCACTTTCCTTCAGCGA | Primers specific to *IDH1* coding sequence for PCR genotyping *35S::IDH1-GFP* transgenic lines |
| TOM20-2_R | *TOM20-2*  AT1G27390 | CCATGCCAACCCAAGCAACAA | Primers specific to *TOM20-2* coding sequence for PCR genotyping *35S::GFP-TOM20-2* transgenic lines |
| TOM20-3_R | *TOM20-3*  AT3G27080 | TCCAAGCAACAACACCAATGGC | Primers specific to *TOM20-3* coding sequence for PCR genotyping *35S::GFP-TOM20-3* transgenic lines |
| ACT2_F | *ACTIN2*  AT3G18780 | GTTCTCTCCTTGTACGCCAG | PCR primer for *ACTIN2* –positive control reference gene |
| ACT2_R |  | TCTTTGCTCATACGGTCAGC |  |
| qPDC1_F | *PDC1*  *AT4G33070* | CTAGTGAAACCGCTGTGATTG | qRT-PCR primer for *PDC1* |
| qPDC1_R |  | CTTCTCTGGTGATGCCTGTG |  |
| qADH1_F | *ADH1*  *AT1G77120* | TGCAGAAGGTGCTAGAATCG | qRT-PCR primer for *ADH1* |
| qADH1_R |  | GTCTTTCGGGTTCACACACT |  |
| qPGB1_F | *PGB1*  *AT2G16060* | GCCAGCCATTCTAAATACGG | qRT-PCR primer for *PGB1* |
| qPGB1_R |  | GCCTCCTTTATCGTCTCCAA |  |
| qPEX4_F | *PEX4 AT5G25760* | CTGCGACTCAGGGAATCTTCTA | qRT-PCR primer for *PEX4* – reference gene |
| qPEX4_R |  | TTGTGCCATTGAATTGAACCC |  |

The Gateway® attB recombination sequences within primers are specified as underlined text. Any additional bases included to maintain reading frames are given as bold and underlined. The BamHI (Bam) restriction enzyme recognition sites upstream of the primer sequences are given in green text.
